# ﻿A new genus and a new species of wasp-mimicking Harpactorini (Hemiptera, Heteroptera, Reduviidae, Harpactorinae), with an updated key to the Neotropical genera

**DOI:** 10.3897/zookeys.1152.96058

**Published:** 2023-03-09

**Authors:** Hélcio R. Gil-Santana, Jader Oliveira

**Affiliations:** 1 Laboratório de Diptera, Instituto Oswaldo Cruz, Av. Brasil, 4365, 21040-360, Rio de Janeiro, RJ, Brazil Laboratório de Diptera, Instituto Oswaldo Cruz Rio de Janeiro Brazil; 2 Universidade de São Paulo, Faculdade de Saúde Pública, Laboratório de Entomologia em Saúde Pública, São Paulo, SP, Brazil Universidade de São Paulo São Paulo Brazil; 3 Laboratório de Parasitologia, Universidade Estadual Paulista “Julio de Mesquita Filho”, Faculdade de Ciências Farmacêuticas UNESP/FCFAR, Rodovia Araraquara Jaú, KM 1, 14801-902, Araraquara, SP, Brazil Universidade Estadual Paulista “Julio de Mesquita Filho” Araraquara Brazil

**Keywords:** *Graptocleptes*, *Hiranetis*, *Parahiranetis*, sexual dimorphism, wasp mimicry

## Abstract

*Quasigraptocleptesmaracristinae***gen. nov.**, **sp. nov.** (Hemiptera, Heteroptera, Reduviidae, Harpactorinae, Harpactorini) is described based on male and female specimens from Brazil. Photographs and comments about the syntypes of *Myocorisnigriceps* Burmeister, 1835, *Myocorisnugax* Stål, 1872, *Myocoristipuliformis* Burmeister, 1838 and *Xystonyttusichneumoneus* (Fabricius, 1803) are presented. The intra-specific variability and sexual dimorphic characteristics among specimens of *Q.maracristinae***sp. nov.** are recorded. General characteristics of *Hiranetis* Spinola, 1837, *Graptocleptes* Stål, 1866, *Quasigraptocleptes***gen. nov.** and *Parahiranetis* Gil-Santana, 2015, which seem to be closer genera, are compared, including those of the male genitalia of some species. A key to the species of *Myocoris* Burmeister, 1835, and an updated key to Neotropical wasp-mimicking Harpactorini genera are provided.

## ﻿Introduction

The subfamily Harpactorinae has the largest number of genera and species of Reduviidae (Hemiptera, Heteroptera) and is represented by the tribes Apiomerini and Harpactorini in the Neotropical region (Gil-Santana et al. 2015). Harpactorini is the most diverse group within Reduviidae with about 52 genera in the Neotropics (Forero 2011; Gil-Santana 2015; Gil-Santana et al. 2015, 2017; Forero and Mejía-Soto 2021). Several taxa of this tribe are recognized as being involved in mimicry systems with Hymenoptera, resembling bees or wasps in general body and wing coloration as well as characteristics of physical proportions (Champion 1899; Haviland 1931; Elkins 1969; Maldonado and Lozada 1992; Hogue 1993; Leathers and Sharkey 2003; Gil-Santana 2008, 2015, 2016, 2022; Gil-Santana et al. 2013, 2015, 2017; Castro-Huertas and Forero 2021). Maldonado and Lozada (1992) presented a key to Neotropical wasp-mimicking Harpactorinae genera, which in their view helps to quickly sort out specimens from unidentified material, although this is a somewhat artificial way of grouping genera. Gil-Santana (2015) has updated their key, including two additional genera to it, *Coilopus* Elkins, 1969 and *Parahiranetis* Gil-Santana, 2015. Among the genera presented in this key, while *Coilopus* is regarded as mimicking species of Vespidae (Elkins 1969; Forero and Giraldo-Echeverry 2015), the remaining genera are considered as mimetic of Braconidae and/or Ichneumonidae and in need of a comprehensive revision in order to clarify their systematics (Gil-Santana et al. 2015). For most of the genera included in this group, more recent works have described or redescribed them or at least some of their species, providing figures and more comprehensive descriptions, allowing a better knowledge of their characteristics as follows: *Acanthischium* Amyot & Serville, 1843 (Castro-Huertas and Forero 2021), *Coilopus* (Elkins 1969; Gil-Santana and Forero 2009; Forero and Giraldo-Echeverry 2015), *Graptocleptesbicolor* (Burmeister, 1838) (Gil-Santana et al. 2013), *Hiranetis* Spinola, 1837 (Gil-Santana 2016), *Neotropiconyttus* Kirkaldy, 1909 (Maldonado and Lozada 1992), and *Parahiranetis* Gil-Santana, 2015 (Gil-Santana 2015; Gil-Santana et al. 2017). However, in regard to *Myocoris* Burmeister, 1835 and *Xystonyttus* Kirkaldy, 1909, there are no published images of their species, and the information about their characteristics are restricted to short original descriptions or their mentioning in diagnosis or keys (e.g., Stål 1872; Maldonado and Lozada 1992). It is noteworthy that among other genera of Harpactorini, such as *Zelus* Fabricius, 1803, there are some species that are considered as being similar to braconids, e.g., *Zelusvespiformis* Hart, 1987 and *Z.errans* Fabricius, 1803, while most of the other numerous species of the same genus are not (Zhang et al. 2016). Therefore, only those genera mentioned before, in which all or most of the known included species have been recognized as wasp-mimetic (Maldonado and Lozada 1992; Gil-Santana 2015), will be considered as such for the purpose of our study.

Most authors have only mentioned or given attention to the pattern of yellowish or straw-colored hemelytra with a median transverse black band, in relation to the alleged mimicry between Harpactorini and certain Ichneumonidae and Braconidae, as models (Champion 1899; Haviland 1931; Maldonado and Lozada 1992; Hogue 1993; Leathers and Sharkey 2003; Hespenheide 2010; Zhang et al. 2016). However, Gil-Santana (2015) and Gil-Santana et al. (2017) emphasized that other wasp-mimicking Harpactorini, like *Parahiranetissalgadoi* Gil-Santana, 2015, show a pattern of darkened to reddish general coloration with yellowish ‘pterostigmata’ on the hemelytra, which is similar to the similar coloration exhibited in the forewings by several other species of Ichneumonidae and Braconidae. This pattern was also observed for instance in *Graptocleptesbicolor* (Burmeister, 1838), *G.haematogaster* (Stål, 1860), and an undescribed species of *Hiranetis* Spinola, 1840 (Gil-Santana 2016; Gil-Santana et al. 2017). Yet, in some other species of wasp-mimicking Harpactorini, the hemelytra are almost or completely dark, such as in *Hiranetisatra* Stål, 1872 (Gil-Santana 2016). Another common feature among all these species with a darkened general coloration on the hemelytra, with or without yellowish ‘pterostigmata’, is the presence of pale bands on the middle and hind femora (Gil-Santana 2015, 2016).

A variable degree of intraspecific variation in color, occasionally at extreme range, was documented in many harpactorines (e.g., Stål 1872; Champion 1899; Gil-Santana 2008, 2022; Zhang et al. 2016; Forero and Mejía-Soto 2021), including in some wasp-mimicking Harpactorini (Champion 1899; Gil-Santana et al. 2013, 2017). However, at least in the species with the pattern of darkened or blackish hemelytra with yellowish pterostigmata, there is no variation in this pattern. The yellowish pterostigmata are always present (e.g., Gil-Santana et al. 2013, 2017).

Numerous species of Harpactorini have shown sexual dimorphism. In several species belonging to *Zelus* Fabricius, 1803, for example, males and females differ drastically in size, body configuration, and coloration (Zhang et al. 2016). In *Acanthischium* Amyot & Serville, 1843 the pattern of coloration was found as being sexually dimorphic in most species of the genus (Castro-Huertas and Forero 2021). In addition to the bigger size and larger abdomen of females, males in several genera have larger eyes and/or a basally thickened antennal basiflagellomere. The latter has been considered to be among the diagnostic features at the generic level (Stål 1872; Champion 1899; Martin-Park et al. 2012; Gil-Santana et al. 2013, 2017; Gil-Santana 2016). However, sexual dimorphism may also be limited to minor differences in coloration and size, as in many species of *Zelus* (Zhang et al. 2016).

The male genitalia has been found to provide useful diagnostic characteristics for distinguishing species within the genera of Harpactorini by diverse authors (e.g. Elkins 1954a, b; Hart 1975, 1986, 1987; Forero et al. 2008; Zhang et al. 2016; Gil-Santana et al. 2017; Castro-Huertas and Forero 2021). Among wasp-mimicking Neotropical Harpactorini, they have been described for *Graptocleptesbicolor* (Burmeister, 1838) (Gil-Santana et al. 2013), *Hiranetisatra* Stål, 1872 (Gil-Santana 2016), *Parahiranetissalgadoi* Gil-Santana, 2015 (Gil-Santana et al. 2017) and species of *Acanthischium* Amyot & Serville, 1843 (Castro-Huertas and Forero 2021). The genera to which the former three species belong, *Graptocleptes* Stål, 1866, *Hiranetis* and, *Parahiranetis* Gil-Santana, 2015, are considered to be closely related to each other (Stål 1872; Champion 1899; Gil-Santana 2015, 2016; Gil-Santana et al. 2017), allowing for important comparisons of several diagnostic traits of male genitalia.

Besides describing a new genus and new species, *Quasigraptocleptesmaracristinae* gen. nov., sp. nov., photographs of the syntypes of *Myocorisnigriceps* Burmeister, 1835, *Myocorisnugax* Stål, 1872, *Myocoristipuliformis* Burmeister, 1838 and *Xystonyttusichneumoneus* (Fabricius, 1803) are provided to record the main characteristics of the genera to which they belong. An improved and updated version of the key to Neotropical wasp-mimicking Harpactorini genera, previously presented by Maldonado and Lozada (1992) and Gil-Santana (2015), is also provided.

## ﻿Materials and methods

**Figures 1–8.**
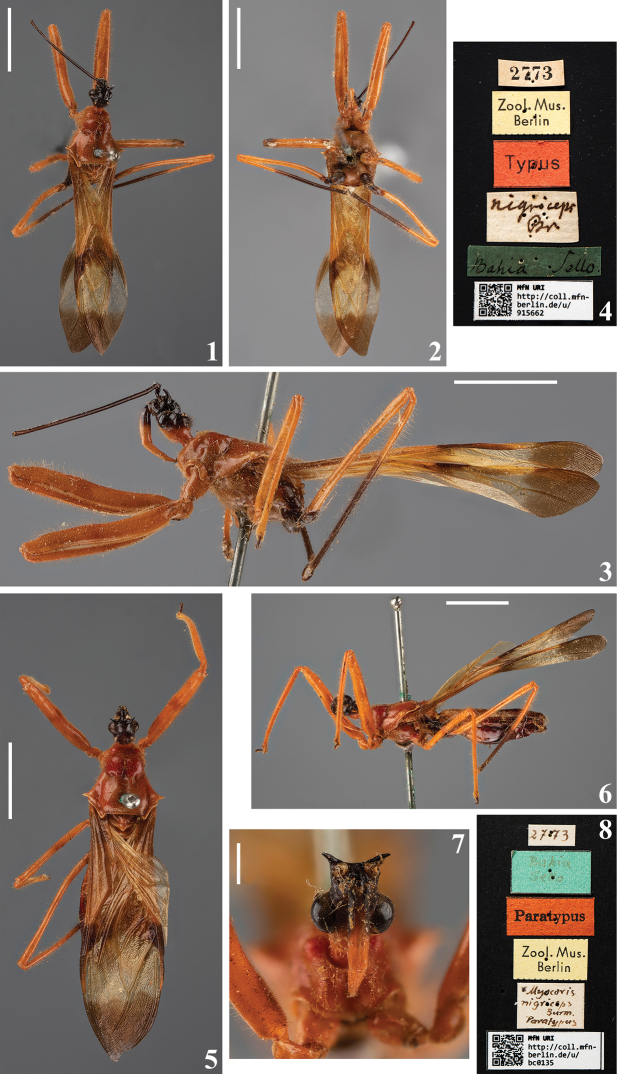
*Myocorisnigriceps* Burmeister, 1835, syntypes deposited in MFNB**1–4** sex not determined, catalog number MfN URI http://coll.mfn-berlin.de/u/915662**1** dorsal view **2** ventral view **3** lateral view **4** labels **5–8** female, catalog number MfN URI http://coll.mfn-berlin.de/u/bc0135**5** dorsal view **6** lateral view **7** head, anterior view **8** labels. The copyright of these images is property of the MFNB. Scale bars: 5.0 mm (**1–3, 5, 6**); 1.0 mm (**7**).

**Figures 9–12.**
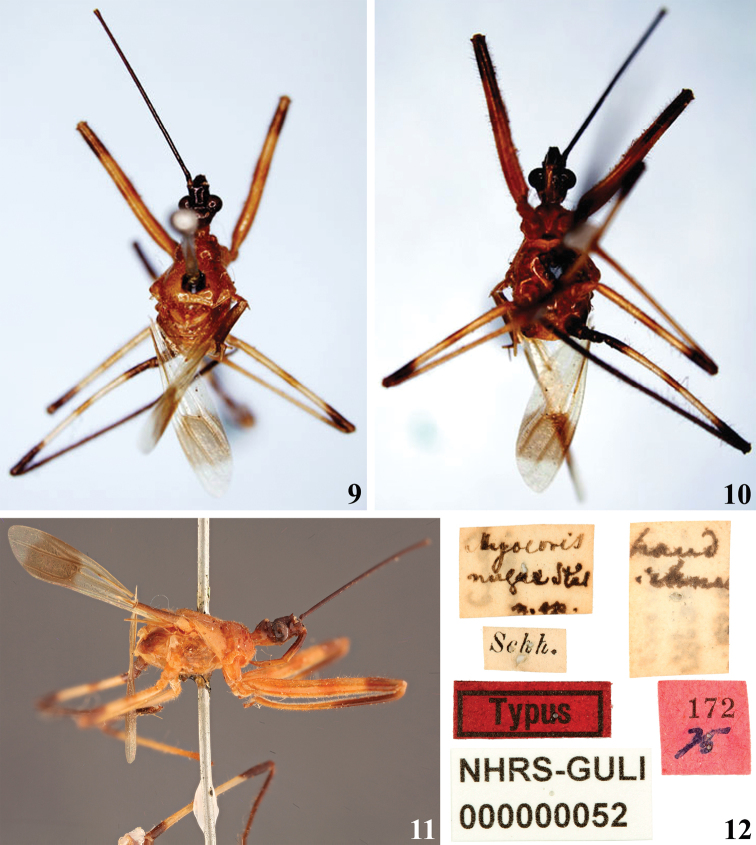
*Myocorisnugax* Stål, 1872, syntype, sex not determined, deposited in NHRS, catalog number NHRS-GULI000000052, photographs provided by Gunvi Lindberg, 2022 Naturhistoriska riksmuseet. Made available by the Swedish Museum of Natural History under Creative Commons Attribution 4.0 International Public License, CC-BY 4.0, https://creativecommons.org/licenses/by/4.0/legalcode**9** dorsal view **10** ventral view **11** lateral view **12** labels.

**Figures 13–16.**
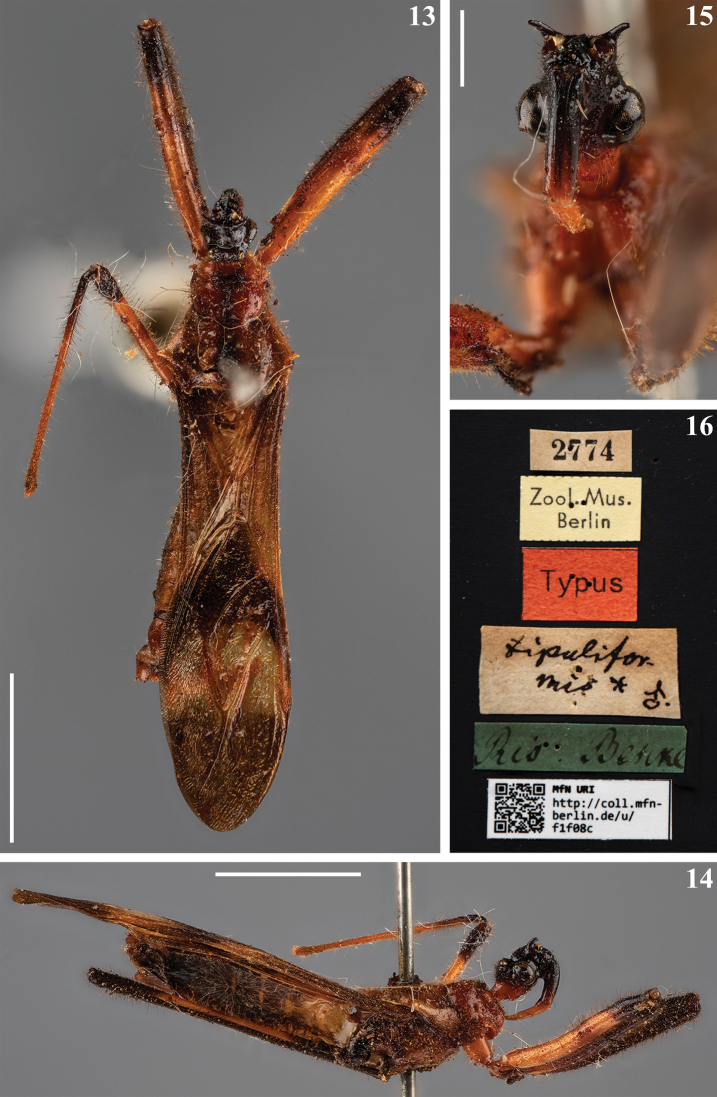
*Myocoristipuliformis* Burmeister, 1838, male syntype deposited in MFNB, catalog number MfN URI http://coll.mfn-berlin.de/u/f1f08c**13** dorsal view **14** lateral view **15** head, anterior view **16** labels. The copyright of these images is property of the MFNB. Scale bars: 5.0 mm (**13, 14**); 1.0 mm (**15**).

Syntypes of *Myocorisnigriceps* Burmeister, 1835 (catalog numbers MfN URI http://coll.mfn-berlin.de/u/915662 and MfN URI http://coll.mfn-berlin.de/u/bc0135) and *Myocoristipuliformis* Burmeister, 1838 (catalog number MfN URI http://coll.mfn-berlin.de/u/f1f08c) deposited in the Hemimetabola Collection of the Museum für Naturkunde Berlin, Leibniz Institute for Evolution and Biodiversity Science, Berlin, Germany (**MFNB**), were directly examined by the first author (HRG-S), while their images (Figs 1–8, 13–16) were provided by Birgit Jaenicke and the copyright of these images is property of the MFNB. Images of a syntype [sex not determined] of *Myocorisnugax* Stål, 1872 (Figs 9–12), deposited in the Swedish Museum of Natural History, Stockholm, Sweden (**NHRS**) were provided by Gunvi Lindberg and are copyright (2022) of the NHRS. The photographs of the female syntypes of *Zelusichneumoneus* Fabricius, 1803, deposited in the Natural History Museum of Denmark (**ZMUC**), Copenhagen, Denmark, were taken by Sree Gayathree Selvantharan (Figs 101–103, 106–108) and Anders Alexander Illum (Figs 104, 105), and provided by Lars Vilhelmsen.

The original photographs were cropped, their lighting and contrast slightly adjusted but without modifying their characteristics, while the numbered scales of Figs 1–3, 5–7, 13–15, 101, 102, 104–107 were reduced or modified to similar simple scale bars in order to standardize them.

Photographs of paratypes of *Quasigraptocleptesmaracristinae* sp. nov. (Figs 20–24, 26, 34, 36, 38, 39, 42, 47–50, 55–72, 89–97) were taken by the second author (JO) using a stereoscope microscope (Leica 205A) with a digital camera. Scanning electron microscopy images (Figs 27, 28, 30–33, 35, 37, 40, 41, 43–46, 51–54, 73–76, 98–100) were also obtained by JO. Two males and a female paratypes of *Quasigraptocleptesmaracristinae* sp. nov. were cleaned in an ultrasound machine. Subsequently, the samples were dehydrated in alcohol, dried in an incubator at 45 °C for 20 min, and fixed in small aluminum cylinders with transparent glaze. Sputtering metallization was then performed on the samples for 2 min at 10 mA in an Edwards sputter coater. After this process, the samples were studied and photographed using a high-resolution field emission gun scanning electron microscope (SEM; JEOL, JSM-6610LV), similarly as described by Rosa et al. (2010, 2014). All remaining figures were produced by the first author (HRG-S). The fixed adults, microscopic preparations, and genitalia were photographed using digital cameras (Nikon D5600 with a Nikon Macro Lens 105 mm and Sony DSC-W830). Drawings were made using a camera lucida. For clarity, the vestiture (setation) was omitted in the ink drawings of Figs 25, 29, 77. Images were edited using Adobe Photoshop CS6.

**Figures 17–20.**
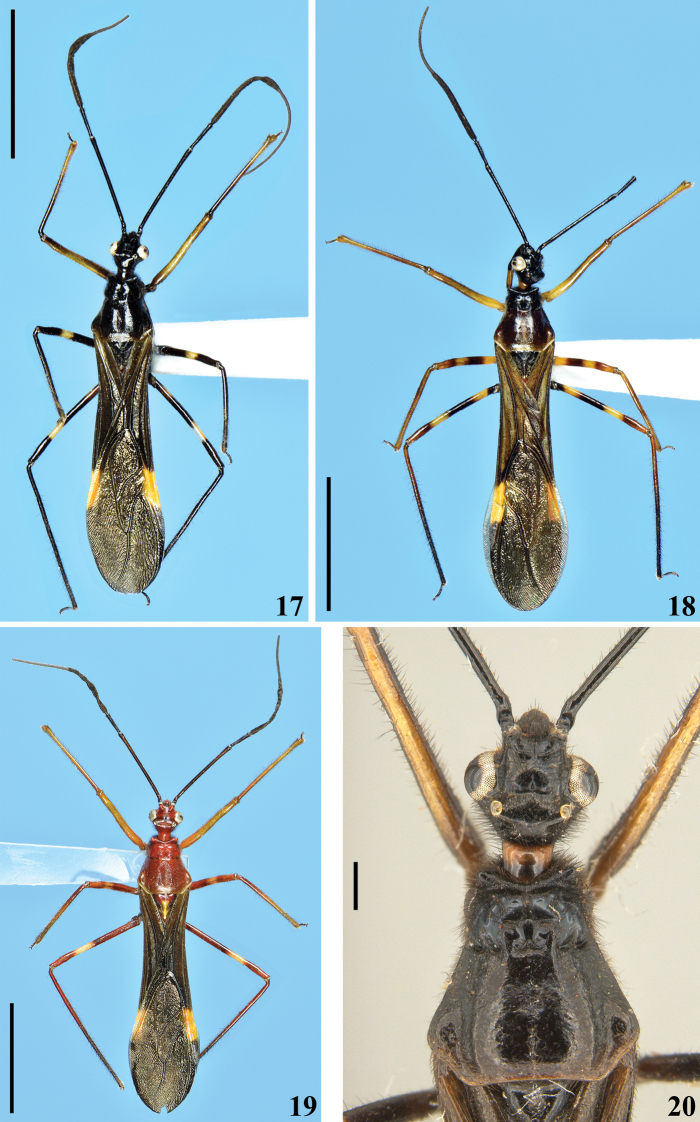
*Quasigraptocleptesmaracristinae* gen. nov., sp. nov., males, dorsal view **17** holotype **18–20** paratypes **20** head and pronotum. Scale bars: 5.0 mm (**17–19**); 0.5 mm (**20**).

Observations were made using a stereoscope microscope (Zeiss Stemi) and a compound microscope (Leica CME). Measurements were made using a micrometer eyepiece. The total length of the head was measured excluding the neck, for better uniformity of this measurement. Dissections of the male genitalia were made by first removing the pygophore from the abdomen with a pair of forceps and then clearing it in 20% NaOH solution for 24 hours. The dissections were carried out on the genitalia of different males presenting the range of color variation recorded among them (e.g., Figs 17–19). The dissected structures were studied and photographed in glycerol.

General morphological terminology mainly follows Schuh and Weirauch (2020). Currently, there is a lack of consensus about the terminology to be applied to female and male genitalia in Reduviidae (e.g., Rédei and Tsai 2011). Therefore, in order to maintain uniformity with previous works about species of Harpactorini, the terminology of the male and female genitalia structures generally follows Gil-Santana et al. (2013, 2017) and Gil-Santana (2016).

The type specimens of *Quasigraptocleptesmaracristinae* gen. nov., sp. nov. will be deposited as follows: male holotype, 2 male paratypes and 1 female paratype in the Entomological Collection of the “Museu Nacional da Universidade Federal do Rio de Janeiro”, Rio de Janeiro, Brazil (**MNRJ**); 6 male paratypes and 1 female paratype in the Dr Jose Maria Soares Barata Triatominae Collection (**CTJMSB**) of the São Paulo State University Julio de Mesquita Filho, School of Pharmaceutical Sciences, Araraquara, São Paulo, Brazil.

When describing label data, a slash (/) separates the lines and a double slash (//) different labels, and comments or translations to English of the label data are provided in square brackets ([]). All measurements are in millimeters (mm).

## ﻿Taxonomy

**Subfamily Harpactorinae**

### ﻿Tribe Harpactorini

#### ﻿ Myocoris

Taxon classificationAnimaliaHemipteraReduviidae

Burmeister, 1835

E789C3CD-DF29-5E1D-A35F-A8B790515B96

Myocoris Burmeister, 1835: 221 [key], 226 [description], 1838: 104 [diagnostic characteristics]; Stål 1859: 367 [key], 370 [diagnostic characteristics], 1866: 294 [key], 1872: 69 [key], 83 [catalog]; Walker 1873a: 49 [key], 63 [key], 1873b: 128 [catalog]; Lethierry and Severin 1896: 178 [catalog]; Wygodzinsky 1949: 42 [catalog]; Putshkov and Putshkov 1985: 52 [catalog]; Maldonado 1990: 236 [catalog]; Maldonado and Lozada 1992: 165 [key]; Forero 2011: 15 [checklist]; Gil-Santana 2015: 36 [key], 2016: 92 [citation]; Gil-Santana et al. 2017: 41 [citation]. Type species: Myocorisnigriceps Burmeister, 1835: 226, by subsequent designation, Wygodzinsky 1949: 42.
Cosmonyttus Stål 1866: 295 [key, in part, including M.nigriceps]; synonym proposed by Kirkaldy 1909: 388. Type species: Myocorisnigriceps Burmeister, 1835: 226, by monotypy.

##### Morphological remarks.

Head cylindrical, elongated, with sparse thin setae on ventral and anteocular portions; postantennal spines short, curved forward, apices acute or somewhat rounded. Legs: fore femora thickened, narrowing at apices and somewhat curved at basal half; fore tibia curved at apical third; middle and hind legs elongated, slender; hind femora curved approximately at basal half.

Burmeister (1835) described *Myocoris* and two new species included in this genus: *M.nigriceps* and *M.braconiformis*. In his subsequent paper, “Some account of the Genus *Myocoris*…” (Burmeister 1838), he considered ten species as included in the genus, some of which were described as new in this occasion, such as *M.tipuliformis*. While several of these ten species were transferred to other genera or considered as synonyms of other species (Maldonado 1990), Stål (1872) described *M.nugax* and maintained only two other species included in *Myocoris*: *M.nigriceps* and *M.tipuliformis*. With exception of Walker (1873b), who considered 26 species as belonging to *Myocoris*, all other subsequent authors (Lethierry and Severin 1896; Wygodzinsky 1949; Putshkov and Putshkov 1985; Maldonado 1990) followed Stål (1872). The three species currently included in *Myocoris* were described from Brazil and accordingly with Stål (1872) may be separated by the following key:

**Table:** 

1	Humeral angles acutely spined (Figs 5, 13)	**2**
–	Humeral angles not acute, rounded (Fig. 9)	***M.nugax* Stål, 1872**
2	Only the basal portion of the first visible labial segment blackish (Fig. 3)	***M.nigriceps* Burmeister, 1835**
–	First visible labial segment entirely blackish (Fig. 14)	***M.tipuliformis* Burmeister, 1838**

Stål (1872) stated that *M.nigriceps* and *M.tipuliformis* were very related and similar species, while *M.nugax* although similar to *M.tipuliformis*, differed from the latter by the somewhat shorter neck, shorter postantennal spines, and humeral angle without acute prominence. Another difference in coloration between *M.nigriceps* and *M.tipuliformis* is in their legs. In *M.tipuliformis* the apical portion of the femora and basal portion of tibiae are blackish, while in *M.nigriceps* these portions are pale.

#### ﻿ Myocoris nigriceps

Taxon classificationAnimaliaHemipteraReduviidae

Burmeister, 1835

21A8052F-9722-52A5-A9FC-8580814D0B95

Figs 1–8

Myocoris nigriceps Burmeister, 1835: 226 [description], 1838: 105 [redescription]; Stål 1859: 370 [citation], 1872: 83 [catalog]; Walker 1873b: 128 [catalog]; Lethierry and Severin 1896: 178 [catalog]; Wygodzinsky 1949: 42 [catalog]; Maldonado 1990: 236 [catalog].

##### Type material examined.

*Myocorisnigriceps*. ***Syntype*** [sex not determined; abdomen absent]: [printed label]: 2773 // [printed label]: Zool. Mus. / Berlin // [printed red label]: Typus // [handwritten label]: *nigriceps* / Br // [handwritten green label]: Bahia Sello. // [printed label]: [at left side]: QR CODE, [at right side]: MfN URI / http://coll.mfn- / berlin.de/u/ / 915662; **Female syntype**: [handwritten label]: 2773 // [printed label]: Zool. Mus. / Berlin // [printed red label]: Paratypus // [handwritten label]: *Myocoris* / *nigriceps* / Burm. / Paratypus // [handwritten green label]: Bahia / Sello // [printed label]: [at left side]: QR CODE, [at right side]: MfN URI / http://coll.mfn- / berlin.de/u/ / bc0135 (MFNB).

In the Hemimetabola Collection of the Museum für Naturkunde Berlin, Leibniz Institute for Evolution and Biodiversity Science, Berlin, Germany (MFNB) there are two type specimens of *M.nigriceps*. A specimen without abdomen, and sex not determined, labeled as “Typus”, catalog number MfN URI http://coll.mfn-berlin.de/u/915662 (Figs 1–4), and a female labeled as “paratypus”, catalog number MfN URI http://coll.mfn-berlin.de/u/bc0135 (Figs 5–8). Both of them are considered here as syntypes, following Art. 73.2 of the ICZN.

#### ﻿ Myocoris nugax

Taxon classificationAnimaliaHemipteraReduviidae

Stål, 1872

69AEB627-46DF-5662-9158-217CA658CD51

Figs 9–12

Myocoris nugax Stål, 1872: 83 [description]; Lethierry and Severin 1896: 178 [catalog]; Wygodzinsky 1949: 42 [catalog]; Maldonado 1990: 236 [catalog].
Xystonyttus nugax (Burm.); Haviland 1931: 150 [wrong combination and authorship; record from Guyana; comments on difficulties of determination: possible misidentification].

##### Note.

Stål (1872) described *M.nugax* based on an unspecified number of female specimens from Brazil and cited “Mus. Holm.” (NHRS) as the depository of the type specimen(s). Currently, only one type specimen of *M.nugax* was found there and it is without its abdomen (G. Lindberg pers. comm.) (Figs 9–11). Consequently, it is not possible to be sure about its gender, but possibly it is a female taking into account that Stål (1872) mentioned only the female of the species in his description. It is considered a syntype accordingly to Art. 73.2 of the ICZN. Photographs of this specimen allow observing the shorter postantennal spine and the humeral angle without acute prominence, as described by Stål (1872) (Figs 9, 11).

#### ﻿ Myocoris tipuliformis

Taxon classificationAnimaliaHemipteraReduviidae

Burmeister, 1838

8C925201-84F4-5FEE-855C-3FD899D519AB

Figs 13–16

Myocoris tipuliformis Burmeister, 1838: 105 [description]; Stål 1859: 370 [citation], 1872: 83 [catalog]; Walker 1873b: 128 [catalog]; Lethierry and Severin 1896: 178 [catalog]; Wygodzinsky 1949: 42 [catalog]; Maldonado 1990: 236 [catalog].
Hiranetis tipuliformis ; Stål 1860: 76 [catalog].

##### Type material examined.

**Male syntype**: [printed label]: 2774 // [printed label]: Zool. Mus. / Berlin // [printed red label]: Typus // [handwritten label]: *tipulifor*- / mis* ♂. // [handwritten green label]: Rio Bescke // [printed label]: [at left side]: QR CODE, [at right side]: MfN URI / http://coll.mfn- / berlin.de/u/ / f1f08c (MFNB).

The male type specimen deposited in the MFNB (Figs 13–15), labeled as “Typus” (Fig. 16), is herewith considered as a syntype, accordingly with Art. 73.2 of the ICZN.

##### Comments.

The characteristics stated by Stål (1872) to separate the species of *Myocoris* were confirmed in their respective type specimens (Figs 1–3, 5–7, 9–11, 13–15). However, it is necessary to study more specimens, with a more profound taxonomical approach, to ascertain their value to this separation, and more importantly, to confirm the validity of these species, such as the case of *M.nigriceps* and *M.tipuliformis*, separated only by color differences, which may be subject of intraspecific variation, as commented above.

#### ﻿ Quasigraptocleptes gen. nov.

Taxon classificationAnimaliaHemipteraReduviidae

D509F845-3D8A-5A76-BBE3-A89A4830215F

https://zoobank.org/9A9CBC12-B6B9-4B67-B9D3-3134D0A9978E

##### Type species.

*Quasigraptocleptesmaracristinae* sp. nov., by present designation.

##### Diagnosis.

*Quasigraptocleptes* gen. nov. can be separated from other genera of wasp-mimicking Harpactorini by the combination of the characteristics presented in the key below, and specially by the postantennal spines, which are strongly curved backwards.

##### Description.

Integument mostly shiny, smooth. ***Head*** gibbous, large, approximately as long as wide across eyes (neck excluded); with sparse long and short, straight or somewhat curved blackish setae; the latter much denser, forming pubescence of long blackish thick setae on postocular portion and gula. Clypeus straight in dorsal view, curved in lateral view. Antenna inserted at level of upper third of eye; scape and pedicel straight with shiny and smooth integument; flagellomeres with opaque integument; basiflagellomere variably curved; in males conspicuously thickened approximately in basal half; distiflagellomere thinner than the other segments and slightly curved. Postantennal spines strongly curved backwards and variably directed medially. Eyes globose, glabrous, projecting laterally, prominent in dorsal view, reaching closer to dorsal margin of head at interocular sulcus slightly behind its midportion; not reaching ventral margin of head, which is far from inferior margin of the eye. Interocular sulcus thin and shallow, curved laterally; just anterior to it, on midline, a small oval fossa, followed anteriorly by a very short thin shallow median sulcus, which sometimes is not evident. Ocelli and portion between them elevated, the former much closer to eyes than to each other. Labium stout, curved, reaching prosternum approximately at proximal part of its distal third; segment II (ﬁrst apparent) thickest and longest, straight, surpassing level of posterior portion of eyes; segment IV shortest, triangular, tapering. Neck thin. ***Thorax*.** Anterior collar inconspicuous; anterolateral angles moderately prominent. Transverse sulcus not very deep, interrupted before middle by a pair of submedian shallow carina. Midlongitudinal sulcus on fore lobe of pronotum shallow or not evident at basal half, deeper at distal half, above transverse sulcus, almost or forming a narrow depression; disc of hind lobe smooth; lateral longitudinal sulci well marked at posterior half to posterior two-thirds of hind lobe of pronotum. Humeral angle moderately elevated, rounded at lateral margin. Scutellum with margins elevated, apex thin, acutely pointed or sometimes rounded at its tip. Mesosternum somewhat elevated laterally, with a median U-shaped carina posteriorly. ***Legs***: coxae globose, slightly constricted apically; femora and tibiae slender, elongate and generally straight. Fore femur shorter than head and pronotum together, slightly thickened at basal portion and somewhat curved at midportion; middle and hind femora slightly dilated subapically and slightly narrower at the portion where submedian distal pale annuli are located; apices of all femora with a pair of lateral small tubercles. Fore and middle tibiae thickened apically, the former more than the latter; at apex of fore tibiae a dorsal spur and a mesal comb. Hemelytra long, surpassing abdomen by about half length of membrane. ***Abdomen***: elongate; spiracles rounded.

##### Distribution.

Brazil, States of Minas Gerais and Paraná.

##### Etymology.

The name of the new genus was composed by the Latin word *quasi*, meaning almost, nearly, like, and *Graptocleptes*, in reference to its apparent proximity to the latter genus. The gender is neutral.

#### ﻿ Quasigraptocleptes maracristinae sp. nov.

Taxon classificationAnimaliaHemipteraReduviidae

0FC73B6A-C963-57CD-9A86-1092C5773A09

https://zoobank.org/83AC7695-DE84-4133-8E8C-1FE932D55CF5

Figs 17–20 , 21–24 , 25–27 , 28–33 , 34–40 , 41–44 , 45–51 , 52–54 , 55–63 , 64–67 , 68–77 , 78–81 , 82–87 , 88–94 , 95–100

##### Type material.

**Brazil**, Minas Gerais, Juiz de Fora Municipality, x. 1997, J. da Silva leg.: 1 male holotype (MNRJ), 2 males, 1 female paratypes (MNRJ), 6 male paratypes (CTJMSB); Paraná, Londrina Municipality, 25.ii.2004, Malaise trap, Rafael Barros leg.: 1 female paratype (CTJMSB).

##### Description.

**Male.** Figs 17–87. Measurements are given in Table 1.

**Table 1.** Measurements (mm) of male type specimens (*n* = 9) of *Q.maracristinae* sp. nov.

	Holotype	Mean	SD	Maximum	Minimum
Body length to tip of hemelytra	12.2	13.45	0.55	14.0	12.2
Body length to tip of abdomen	9.5	10.47	0.47	11.0	9.5
Head length (excluding neck)	1.3	1.42	0.19	1.7	1.0
Anteocular portion length^1^	0.32	0.43	0.08	0.5	0.32
Postocular portion length^1^	0.35	0.44	0.06	0.5	0.35
Head width across eyes	1.4	1.34	0.16	1.5	1.1
Interocular distance (synthlipsis)	0.8	0.8	0.08	0.9	0.6
Transverse width of eye	0.32	0.33	0.02	0.38	0.3
Length of eye	0.5	0.5	0.05	0.6	0.4
Ocellar tubercle width	0.9	0.82	0.09	0.9	0.6
Scape length	3.4	3.43	0.1	3.5	3.2
Pedicel length	1.0	0.97	0.04	1.0	0.9
Basiflagellomere length (*n* = 8)^2^	6.5	5.48	0.43	6.5	5.1
Basiflagell. max. width (*n* = 8)^2^	0.2	0.26	0.07	0.4	0.2
Distiflagellomere length (*n* = 3)^2^	–	1.5	0.00	1.5	1.5
Labial segment II length^1^	0.9	0.95	0.07	1.0	0.8
Labial segment III length^1^	0.8	0.82	0.08	0.9	0.7
Labial segment IV length^1^	0.2	0.21	0.03	0.3	0.2
Pronotum length (at midline)	2.0	2.27	0.11	2.4	2.0
Pronotum maximum width	1.9	2.37	0.19	2.5	1.9
Scutellum length	0.9	0.96	0.05	1.0	0.9
Fore femur length	3.2	3.39	0.16	3.7	3.2
Fore tibia length	3.5	3.6	0.15	4.0	3.5
Fore tarsus length (*n* = 7)^2^	0.4	0.4	0.06	0.5	0.3
Middle femur length	2.6	2.9	0.16	3.1	2.6
Middle tibia length	3.2	3.45	0.21	3.7	3.0
Middle tarsus length	0.5	0.52	0.03	0.6	0.5
Hind femur length	3.9	4.26	0.20	4.5	3.9
Hind tibia length	5.1	5.85	0.36	6.2	5.1
Hind tarsus length	0.5	0.57	0.05	0.65	0.5
Abdomen length^3^	5.3	5.58	0.35	6.5	5.3
Abdomen maximum width	1.2	1.52	0.13	1.7	1.2

^1^ Measured in lateral view; ^2^ Segments or portions broken or missing in some specimens; ^3^ Measured on ventral view, at midline, from anterior margin of sternite II to posterior border of genitalia.

**Figures 21–24.**
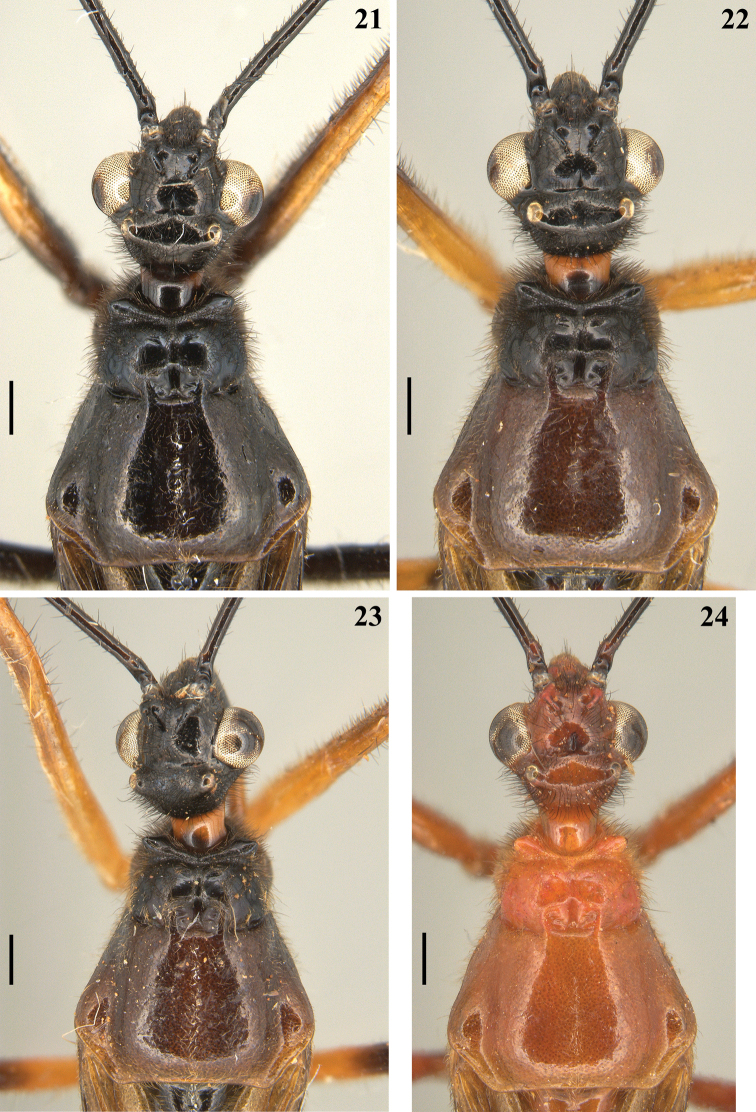
*Quasigraptocleptesmaracristinae* gen. nov., sp. nov., males, head and pronotum, dorsal view. Scale bars: 0.5 mm (**21–24**).

**Figures 25–27.**
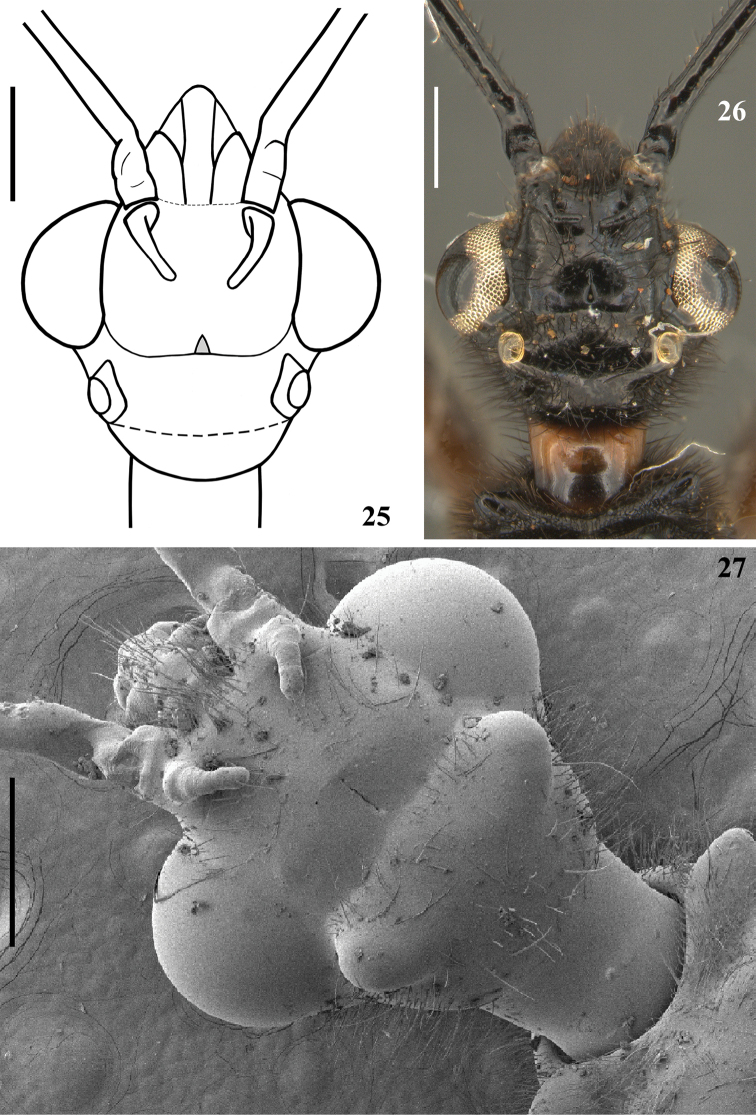
*Quasigraptocleptesmaracristinae* gen. nov., sp. nov., males, head, dorsal view. Scale bars: 0.5 mm (**25–27**).

**Figures 28–33.**
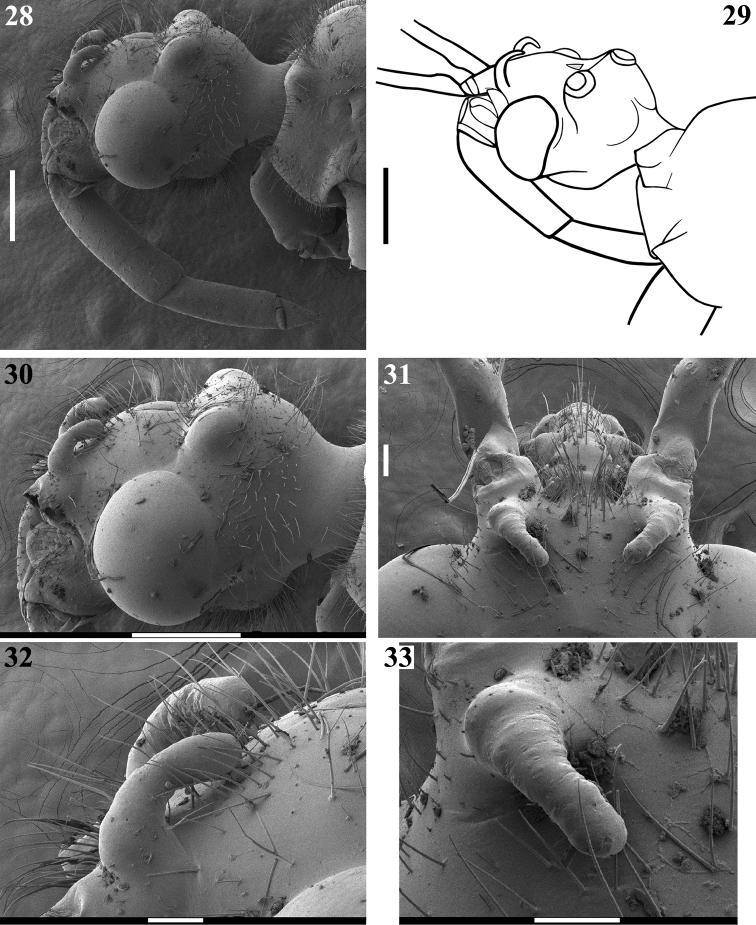
*Quasigraptocleptesmaracristinae* gen. nov., sp. nov., males, head **28–30** posterolateral view **30** upper portion **31** anterior portion, including postantennal spines, dorsal view **32** postantennal spines, laterodorsal view **33** left postantennal spine, dorsal view. Scale bars: 0.5 mm (**28–30**); 0.1 mm (**31–33**).

**Figures 34–40.**
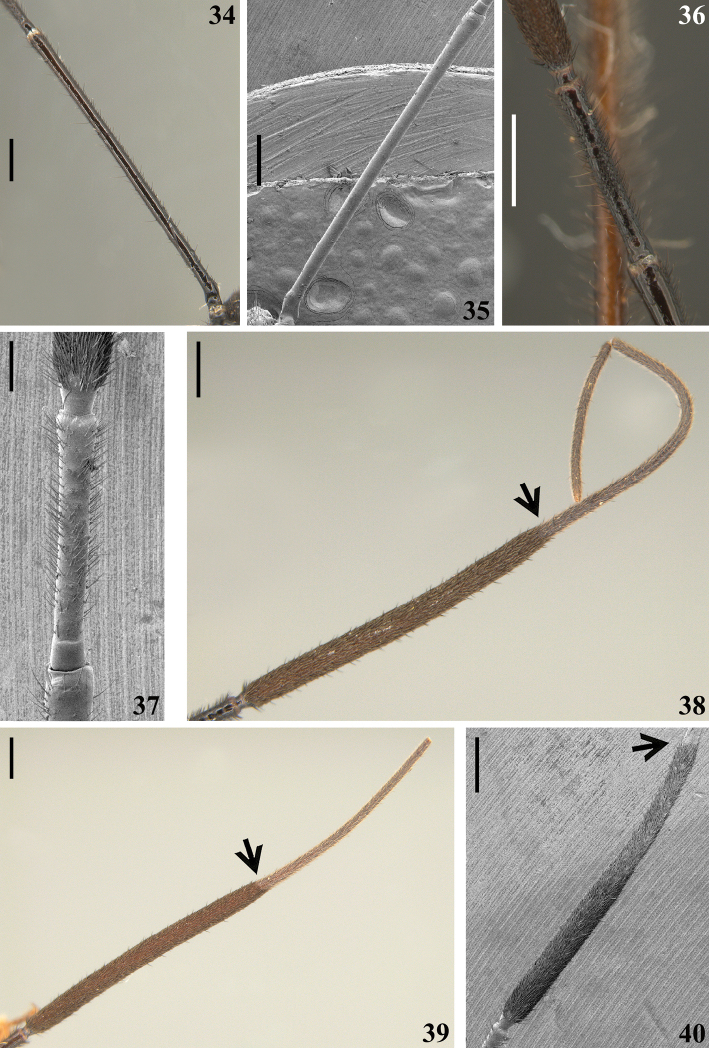
*Quasigraptocleptesmaracristinae* gen. nov., sp. nov., males, antenna, dorsal view **34, 35** scape **36, 37** apex of scape, pedicel and basal portion of basiflagellomere **38–40** arrows point to the portion of clear separation between the thickened and thinner portions of basiflagellomere **38** apex of pedicel and flagellomeres **39** apex of pedicel and basiflagellomere **40** apex of scape and approximately basal half of basiflagellomere. Scale bars: 0.5 mm (**34–36, 38–40**); 0.2 mm (**37**).

**Figures 41–44.**
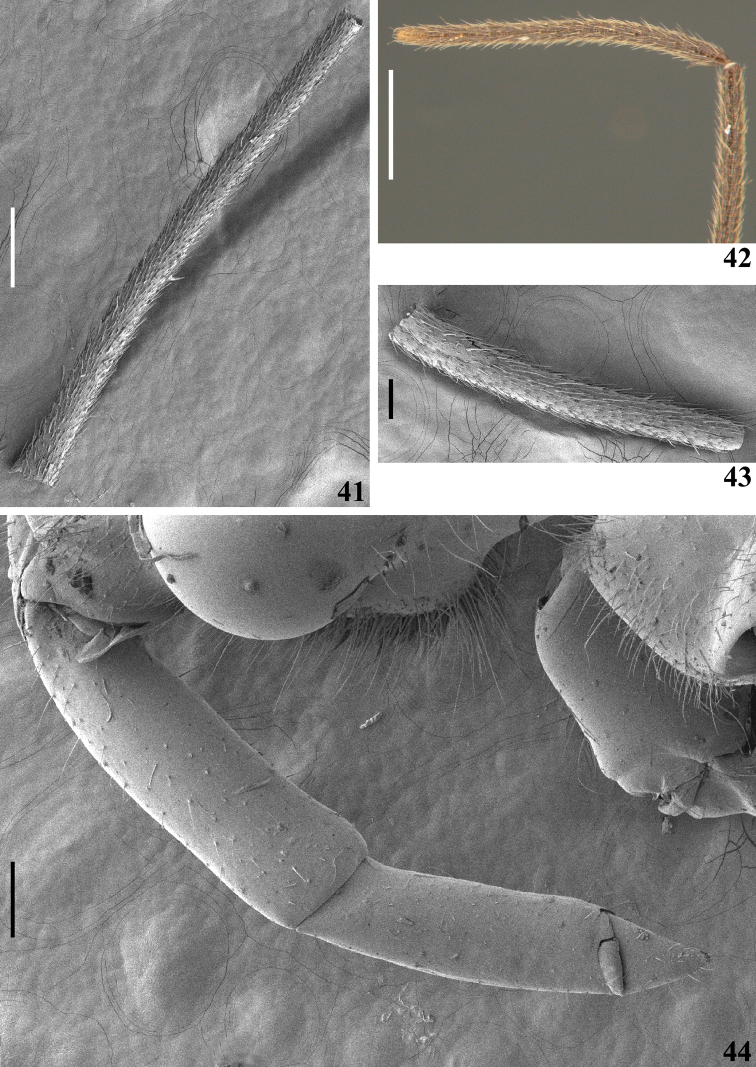
*Quasigraptocleptesmaracristinae* gen. nov., sp. nov., male **41** basiflagellomere, distal half, dorsal view **42** distal portion of basiflagellomere and distiflagellomere, lateral view **43** distiflagellomere, dorsal view **44** lower portion of head and labium, lateral view. Scale bars: 0.5 mm (**42**); 0.3 mm (**41**); 0.2 mm (**44**); 0.1 mm (**43**).

**Figures 45–51.**
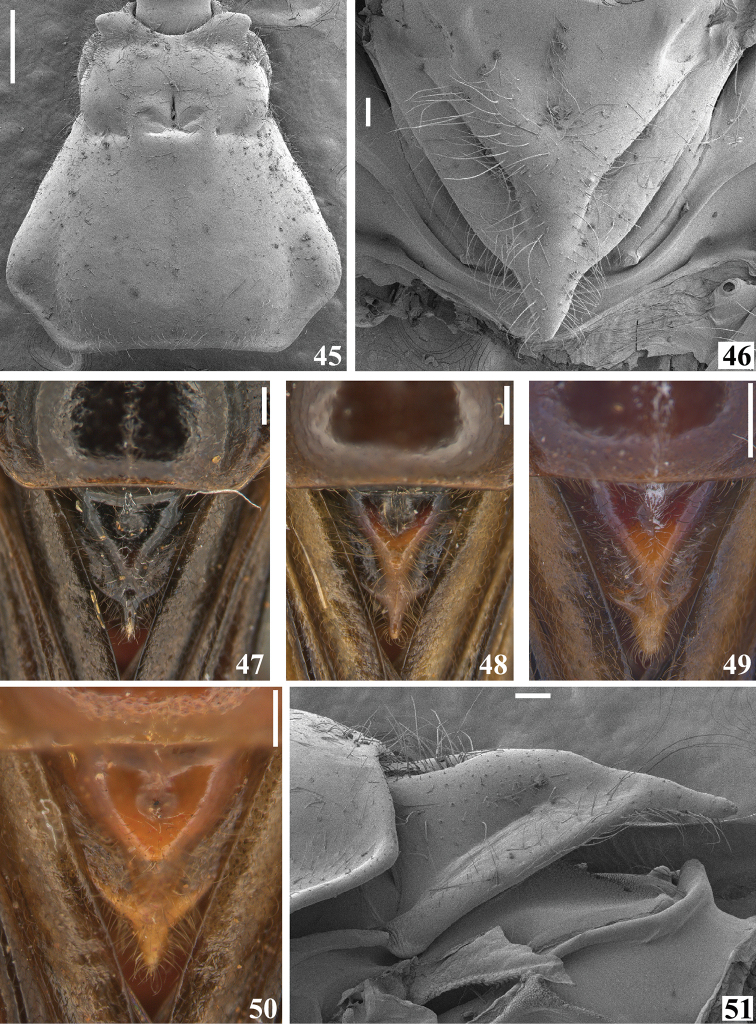
*Quasigraptocleptesmaracristinae* gen. nov., sp. nov., males, thorax **45–50** dorsal view. **45** pronotum **46–51** scutellum **51** lateral view. Scale bars: 0.5 mm (**45, 49**); 0.25 mm (**47, 48, 50**); 0.1 mm (**46, 51**).

**Figures 52–54.**
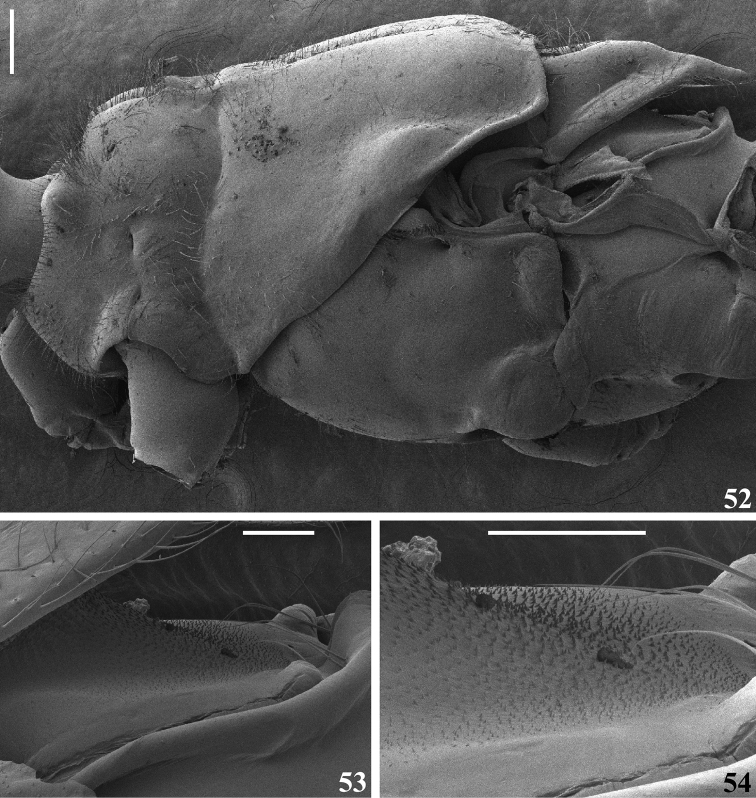
*Quasigraptocleptesmaracristinae* gen. nov., sp. nov., male, thorax, lateral view **52** legs and wings extracted **53, 54** sclerite below scutellum **54** under higher magnification. Scale bars: 0.3 mm (**52**); 0.1 mm (**53, 54**).

**Figures 55–63.**
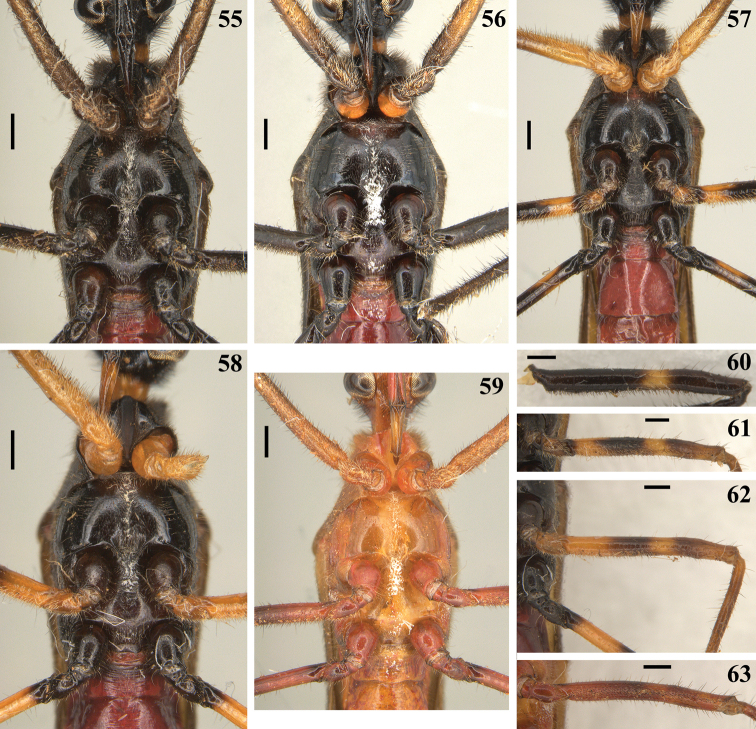
*Quasigraptocleptesmaracristinae* gen. nov., sp. nov., males **55–59** thorax and first visible (s) sternite (s), ventral view **60–63** middle femur **60–62** lateral view **63** ventral view. Scale bars: 0.5 mm (**55–59**); 0.4 mm (**60–63**).

**Figures 64–67.**
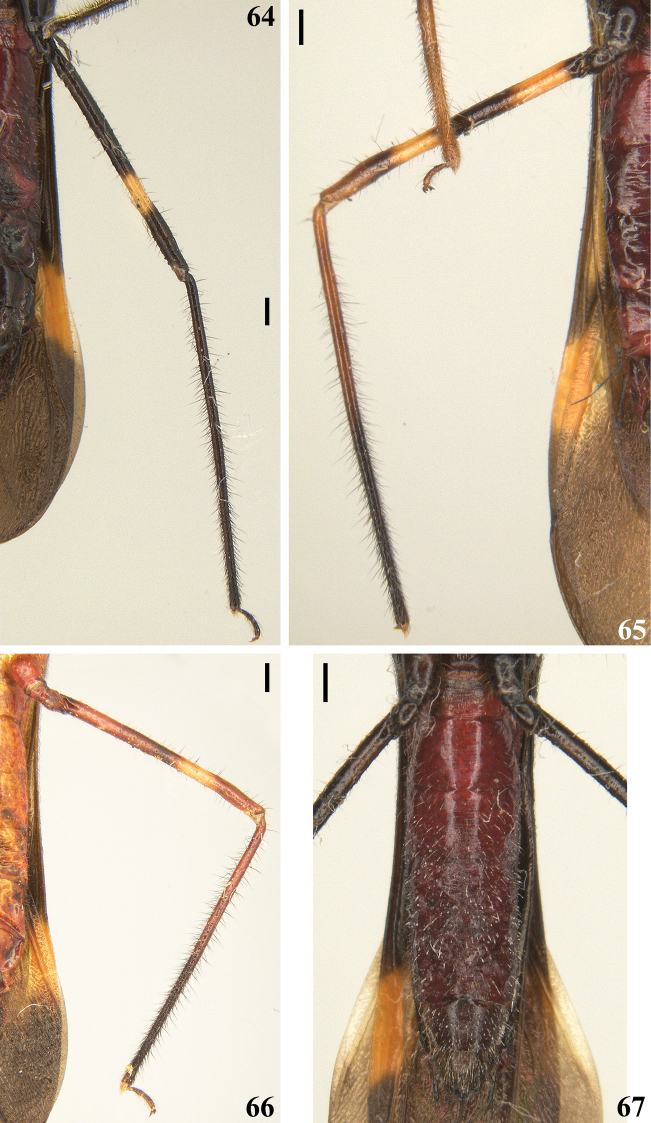
*Quasigraptocleptesmaracristinae* gen. nov., sp. nov., males **64–66** hind leg, lateral view. **67** abdomen, ventral view. Scale bars: 0.5 mm (**64–67**).

**Figures 68–77.**
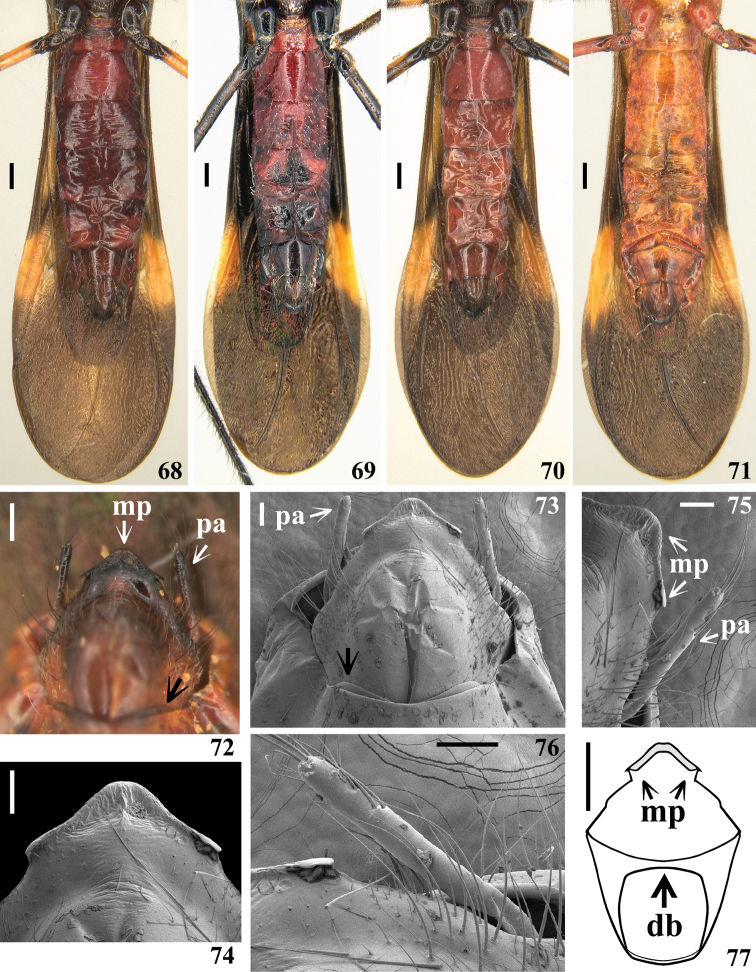
*Quasigraptocleptesmaracristinae* gen. nov., sp. nov., males **68–71** abdomen and apical portion of membrane of hemelytra, ventral view **72–76** ventral view **72, 73** apex of abdomen with pygophore in situ, black arrows point to distal margin of segment VIII **74** medial process of pygophore **75, 76** lateral portion of pygophore and left paramere **77** pygophore without parameres, schematic outline, dorsal view. Abbreviations: **db**: dorsal bridge; **mp**: medial process of pygophore; **pa**: paramere. Scale bars: 0.5 mm (**68–71, 77**); 0.2 mm (**72**); 0.1 mm (**73–76**).

**Coloration**: general coloration black, brownish or reddish (Figs 17–19). ***Head*** black or reddish; antennal segments black, dark brownish or reddish; labium completely dark or with distal half of second visible segment and last segment pale or entirely reddish; neck black, mostly or completely dark yellow or reddish (Figs 17–24, 26, 34, 36, 38, 39, 42). ***Thorax*** completely blackish, dark brown, brownish or reddish, sometimes blackish on fore lobe and brownish on hind lobe; sometimes humeral angles and/or posterior margin of hind lobe of pronotum slightly paler (Figs 17–24). Scutellum with similar coloration of thorax, sometimes with the apex of the process, in variable extension paler or whitish (Figs 17–19, 47–50). Hemelytra generally black with veins concolorous or paler, brownish to dark gray with the veins of the corium darker; a yellowish spot on external and mid-distal portions of corium reaching adjacent part of membrane, especially in basal portion of distal cell of membrane and just posterior to it; in paler specimens, the portion medial to the yellowish spot slightly paler (Figs 17–19). ***Legs*.** All coxae blackish (Fig. 55) or with fore coxae pale on posterior surface (Fig. 56), or completely pale (Figs 57, 58), or all coxae almost or completely pale or reddish (Fig. 59). All trochanters blackish or dark brownish (Figs 55, 56), or fore or the latter and middle trochanters partially or completely pale (Figs 57, 58) or all of them almost or completely pale or reddish (Fig. 59). Femora generally blackish, brownish or reddish. Fore femora frequently with dorsal surface paler, with dark yellowish tinge or generally reddish or dark yellowish in variable extent along the segment (Figs 17–19). Middle and hind femora with one pale or yellowish submedian distal annulus or also with an additional pale or yellowish subbasal or basal annulus (Figs 17, 60, 64, 65); sometimes the subbasal annulus is fainter, darker and/or incomplete, and as such, only evident on hind leg (Fig. 17); in specimens with reddish general coloration, the portion proximal to the submedian distal annuli is sometimes variably darker (Figs 19, 63, 66); additionally, in some specimens with a pair of annuli, the apex of these femora are also paler (Figs 18, 61, 62, 65). Tibiae completely dark or variably pale brownish or reddish on its basal or distal portion, or almost completely or completely pale brownish to pale reddish (Figs 17–19, 64–66). Tarsi in general with a similar coloration to the apex of the respective tibia. ***Abdomen*.** Sternites generally pale reddish, reddish or dark reddish with some or most segments partially or entirely darkened to blackish (Figs 67–71). **Vestiture. *Head*** covered by long and short, straight or somewhat curved blackish setae, which are denser, forming pubescence of long blackish thick setae on postocular portion, even more numerous on gula, and sparser or absent in the area anterior to transverse sulcus (Figs 20–24, 26–28, 30–33). Antenna: scape with sparse short, stiff, slightly curved, dark setae, which become more numerous on mesal surface, approximately in distal two-thirds (Figs 34, 35) and a few longer blackish thin setae scattered along the segment; pedicel, except at glabrous base, covered with numerous short, stiff, obliquely semi-erect dark setae (Figs 36, 37), and a few (about eight to ten) very much thinner isolated elements (interpreted as trichobothria), which are present laterally on basal two-thirds of external surface and dorsally on distal third; thickened portion of basiflagellomere, except at extreme base (which is glabrous), completely covered with short, stiff, dark, adpressed setae, and with scattered stiff, darkened, semi-erect setae and a pubescence formed by longer, very thin, pale setae, which are almost imperceptible in this portion (Figs 38–40); distal (not thickened) portion of basiflagellomere and distiflagellomere covered with dense pubescence formed by short, thin, pale to whitish setae and with scattered short, darkened, stiff, semi-erect setae; the latter somewhat less numerous or not evident on distiflagellomere (Figs 38, 39, 41–43). Labium with scattered and somewhat curved, longer and thinner dark setae (Figs 28, 44). Eyes and ocelli glabrous (Figs 27, 28, 30). Neck almost completely or completely glabrous (Figs 20–24, 26–28, 30). ***Thorax*.** Prothorax covered with very numerous blackish thick setae on fore lobe of pronotum, anterior portions of propleura and hind lobe of pronotum; the latter with sparse long setae at dorsal portion or, almost glabrous, except on midline, where thinner, somewhat shorter and light yellowish to whitish setae form a faint midlongitudinal line on hind lobe; median portion of posterior margin of pronotum with some long thin darkened setae (Figs 20–24, 45, 52). Scutellum with scattered thin dark or pale long setae more or less numerous; sometimes with midlongitudinal line of whitish setae on approximately its basal third, which may be a continuation of the line of whitish setae on hind lobe, or may be present only on the scutellum, while absent on pronotum (Figs 46–52). The pale setae which form the midlongitudinal line on hind lobe of pronotum and/or scutellum are sometimes partially or completely covered by a small amount of white wax-like substance (e.g. Figs 21, 23, 47, 49). Posterior portion of propleura, mesopleura, metapleura and thoracic sterna with less numerous long darkened setae, which are shorter and thinner at center of mesosternum and metasternum (Figs 52, 55–59). In some specimens, there is a group of thin whitish setae basally covered with rounded, flocky patches of white wax-like substance, extending along midline of mesosternum and metasternum and sometimes extending to basal half of first visible sternite (Figs 55, 56, 58, 59). Dorsal sclerite below scutellum covered by numerous minute spiny setae (Figs 53, 54). ***Legs***: coxae with numerous long thin setae on posterior and lateral surfaces, which are less numerous or absent on basal third; fore trochanters densely covered with pale long setae ventrally and with some scattered even longer thinner pale setae; middle and hind trochanters progressively less setose. All femora covered with scattered, long, straight, erect or semi-erect darkened setae and dense, erect, mostly pale, brush-like setae ventrally, which are even more numerous on basal portion and absent on hind femur. All tibiae with scattered long thick blackish setae and covered with shorter dark setae on ventral surface, which become progressively more numerous towards apex, where they also cover lateral and dorsal surfaces. Tarsi covered with shorter dark setae. Hemelytron: clavus and corium generally covered by numerous curved, short, very thin, pale setae, which become much less numerous, adpressed and even shorter on distal half of corium; membrane glabrous. ***Abdomen***: number of setae on sternites varying among individuals, generally with scattered long thin setae, which are light on reddish portions and dark on the blackish segments, and thicker, longer, and also more numerous on parts adjacent to genitalia and on the latter (Figs 67–71). In some specimens, median portion of basal half of first visible sternite with whitish setae covered by white wax-like substance (Figs 56, 59); in one individual, a pair of lateral narrow stripes of sparse whitish setae on distal half of sternite VII, extending to basal exposed portion of pygophore (Fig. 69). ***Structure*.** Antennal basiflagellomere variably slightly curved, from 1.6 to 1.8 times longer than scape, conspicuously thickened approximately in basal half, which is clearly separated in relation to the distal thinner portion (Figs 38–40). Postantennal spines variable in length and thickness among individuals; slightly or strongly directed medially towards their apices, which are blunt to subacute (Figs 20–33). Abdominal segment VIII with only its distal margin of ventral surface visible externally (Figs 72, 73); sclerotized only on ventral portion, which is subrectangular in shape and has both basal and distal margins curved, the latter more than the former, and more prominent laterally.

**Figures 78–81.**
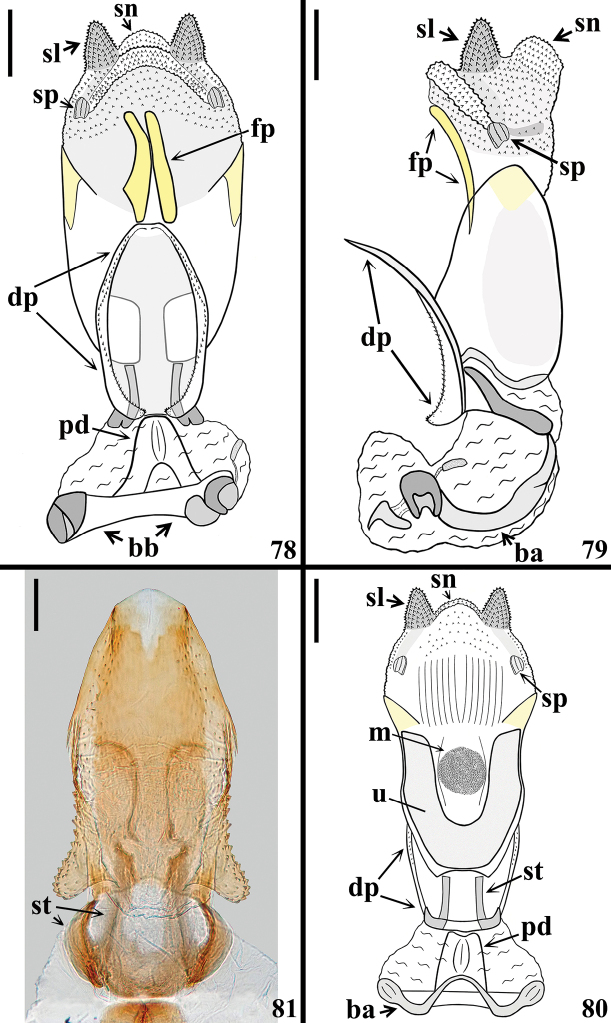
*Quasigraptocleptesmaracristinae* gen. nov., sp. nov., male genitalia **78–80** phallus **78** dorsal view **79** lateral view **80** ventral view **81** dorsal phallothecal plate and struts, dorsal view. Abbreviations: **ba**: basal plate arm; **bb**: basal plate bridge; **dp**: dorsal phallothecal plate; **fp**: flat process of endosoma; **m**: median process of endosoma; **pd**: pedicel; **sl**: sclerotized subtriangular lobe of endosoma wall; **sn**: not sclerotized lobe; **sp**: small sclerotized portion; **st**: strut; **u**: U-shaped process of endosoma. Scale bars: 0.2 mm (**78–80**); 0.1 mm (**81**).

**Figures 82–87.**
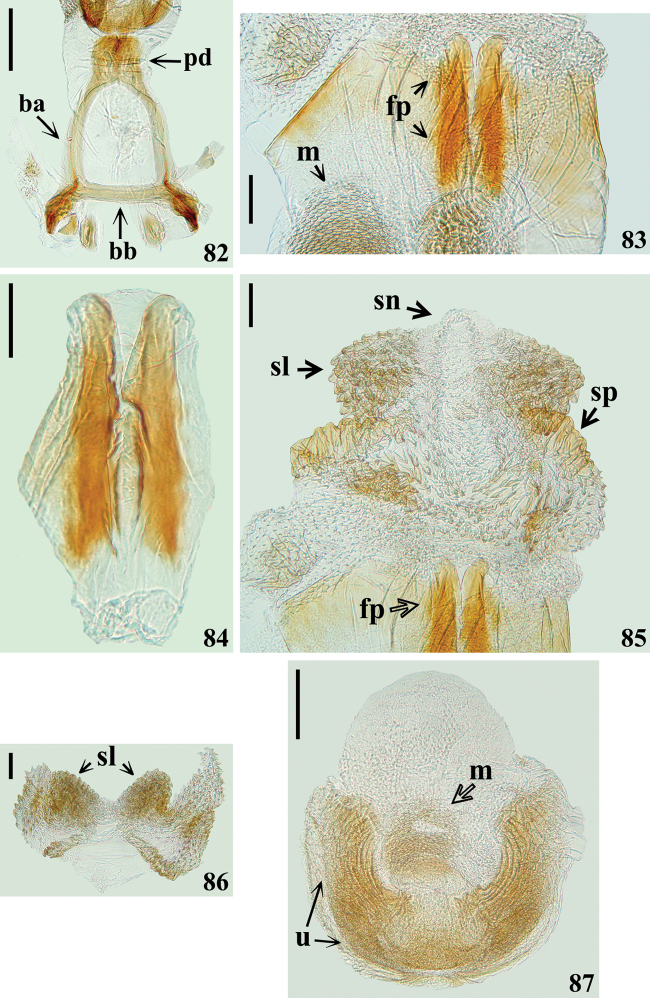
*Quasigraptocleptesmaracristinae* gen. nov., sp. nov., male genitalia **82–86** dorsal view **82** articulatory apparatus **83** phallus, middle portion **84** flat processes of endosoma **85** phallus, apical portion **86** apical subtriangular lobes of endosoma wall; **87** U-shaped and median processes of endosoma, ventral view. Abbreviations: **ba**: basal plate arm; **bb**: basal plate bridge; **fp**: flat process of endosoma; **m**: median process of endosoma; **pd**: pedicel; **sl**: sclerotized subtriangular lobe of endosoma wall; **sn**: not sclerotized lobe; **sp**: small sclerotized portion; **u**: U-shaped process of endosoma. Scale bars: 0.2 mm (**82, 87**); 0.1 mm (**83–86**).

**Male genitalia** (Figs 67–87). Pygophore darkened, blackish; paler or reddish at proximal portion, in paler specimens (Figs 67–72); suboval in ventral view, somewhat enlarged laterally just below the insertions of the parameres (Fig. 73); with an enlarged, somewhat arrow-shaped apex (medial process, mp), in which lateral margins are acutely pointed and the median portion is rounded (Figs 72–75, 77); between anterior and posterior genital openings, a relatively narrow dorsal (transverse) bridge (db) (Fig. 77); ventrolateral margins of exposed portion of pygophore with numerous, long, erect setae (Figs 72, 73, 75, 76). Parameres (pa) symmetrical, rod-like in shape; apices rounded, paler at basal third, becoming darker to blackish in apical half; glabrous in basal two-thirds and with long, stout, dark setae in apical third (Figs 72, 73, 75, 76). Phallus (Figs 78–80): articulatory apparatus with basal plate arms (ba) and basal plate bridge (bb) narrow and forming a subsquared set, except in apical portion, where the arms are curved (Fig. 82); pedicel (pd) (= basal plate extension) short (Figs 78, 80, 82). Dorsal phallothecal plate (dp) weakly sclerotized (Figs 78, 81); subrectangular in dorsal view, somewhat expanded laterally at basal portion and with small acute spines on lateral margins (Figs 78, 79, 81); medially to the latter, a pair of somewhat depressed subrectangular areas on the disc; struts (st) with curved lateral arms, which are thicker basally, and subparallel median arms slightly converging towards apices (Fig. 81). Endosoma wall smooth on basal half, becoming progressively more densely, minutely, spiny towards apex; at distal third: a pair of small more sclerotized lateral portions (sp); an apical pair of prominent sclerotized subtriangular lobes (sl), between which, ventrally, a shallower not sclerotized lobe (sn) (Figs 78–80, 85, 86). The following endosomal processes were observed: 1 - a pair of elongate, parallel, flat, medial and weakly sclerotized processes (fp), wrapped in a smooth portion of endosoma wall, dorsally (Figs 78, 79, 83–85); 2 - a larger U-shaped basal process (u) formed by diffuse thickening (Figs 80, 87); 3 - a median subspherical process (m), situated between the lateral arms of the basal process and formed by a dense grouping of small thickenings (Figs 80, 83, 87).

**Figures 88–94.**
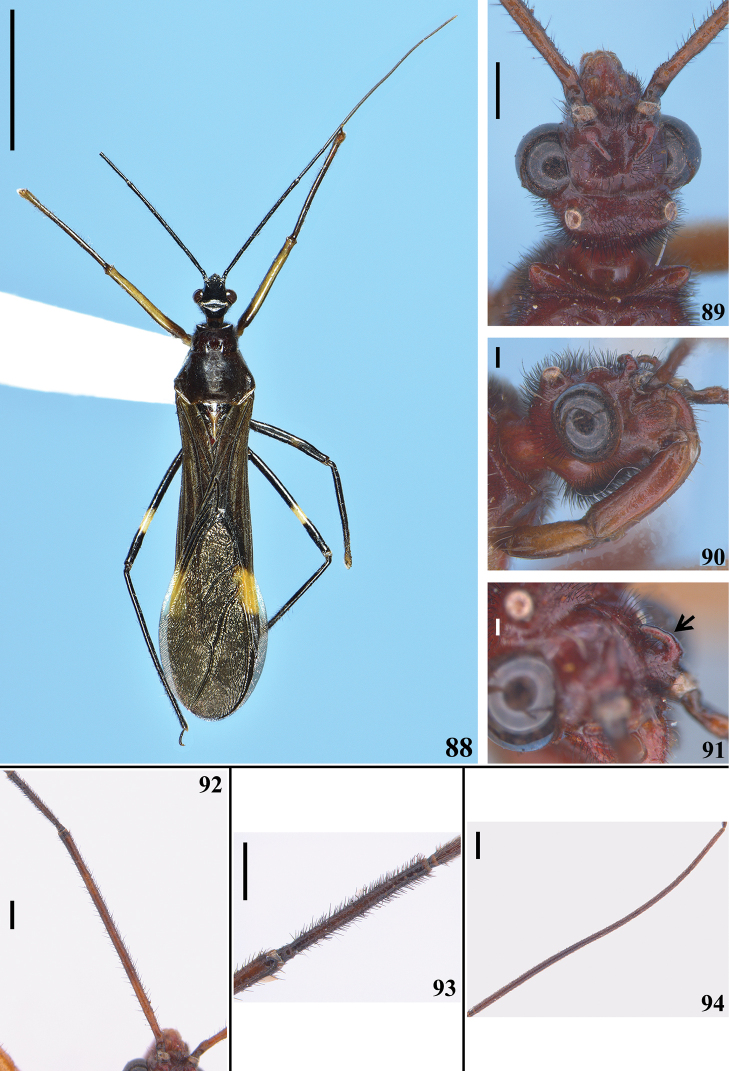
*Quasigraptocleptesmaracristinae* gen. nov., sp. nov., females **88–89** dorsal view. **89** head and anterolateral angles of pronotum **90** head, anterolateral view **91** left postantennal spine (pointed by an arrow), inner surface, lateral view **92–94** antennal segments, dorsal view **92** scape and pedicel **93** apex of scape, pedicel and basal portion of basiflagellomere **94** basiflagellomere. Scale bars: 5.0 mm (**88**); 0.5 mm (**89, 92–94**); 0.2 mm (**90**); 0.1 mm (**91**).

**Figures 95–100.**
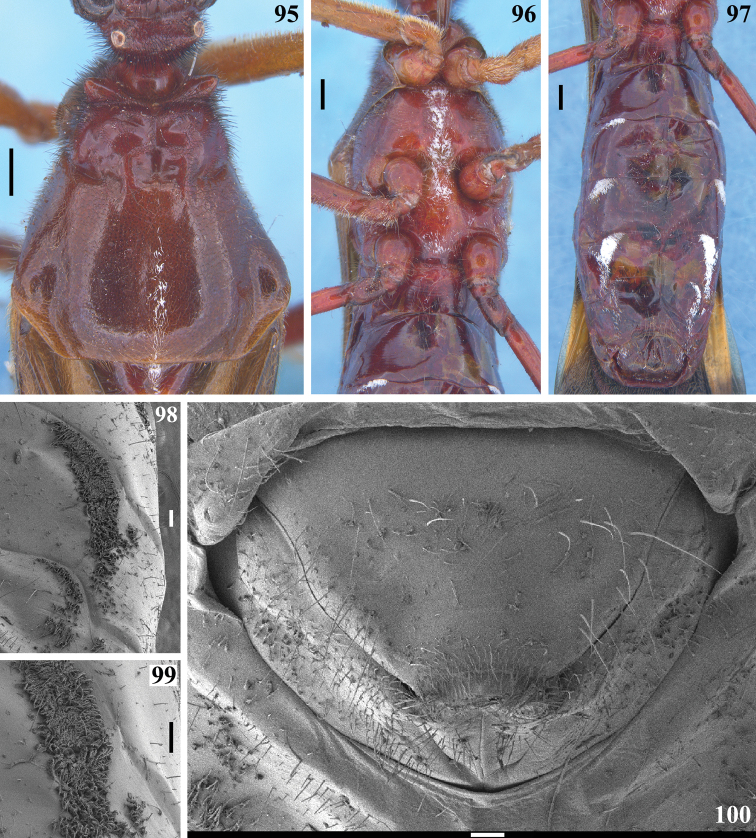
*Quasigraptocleptesmaracristinae* gen. nov., sp. nov., female **95** postocular portion of head, pronotum and basal portion of scutellum, dorsal view **96–99** ventral view **96** thorax and first visible sternites **97** abdomen **98** patches of minute, short, adpressed, thin, whitish setae, covered with a variable amount of white wax on left side of sternites VI–VII **99** main portion of the patch of left side of sternite VI in higher magnification **100** female genitalia, posterior view. Scale bars: 0.5 mm (**95–97**); 0.1 mm (**98–100**).

**Female.** Figs 88–100. Measurements are given in Table 2. Similar to male in general. One specimen with a general dark coloration (Fig. 88) and other with reddish general coloration (Figs 89–97); both with only the submedian distal yellowish annuli on middle and hind femora evident. **Structure. *Head***: basiflagellomere slightly thicker in basal portion (Fig. 94), but much thinner as a whole than that in males, and becoming progressively thinner toward apex, without a clear separation between more or less thickened portions (Fig. 94), uniformly covered with pubescence formed by thin, pale setae (blackish, stiff, adpressed, short setae that completely cover thicker portion in male are absent); approximately 1.2 times longer than scape. Sternites IV–VII with fusiform or elongated patches of minute, short, adpressed, thin, whitish setae, covered with a variable amount of white wax, present on midlateral portions of basal half (sternites IV, V) or, although with more numerous setae on basal portions, extending along the segment on sternites VI and VII; in one specimen, also sparsely on basal portion of the genital tergite 9 (Figs 97–99). External genitalia in posterior view (Fig. 100): tergite 9 with very long, sparse, strong blackish setae at median and lateral portions and numerous shorter, thinner setae at distal margin; tergite 10 with sparse short setae; gonocoxite 8 and gonapophysis 9 with numerous short to somewhat longer setae.

**Table 2.** Measurements (mm) of female type specimens (*n* = 2) of *Q.maracristinae* sp. nov.

	Mean	SD	Maximum	Minimum
Body length to tip of hemelytra	15.3	0.28	15.5	15.1
Body length to tip of abdomen	12.1	0.14	12.2	12.0
Head length (excluding neck)	1.45	0.07	1.5	1.4
Anteocular portion length^1^	0.55	0.62	0.6	0.5
Postocular portion length^1^	0.55	0.62	0.6	0.5
Head width across eyes	1.45	0.07	1.5	1.4
Interocular distance (synthlipsis)	0.8	–	0.8	0.8
Transverse width of eye	0.34	0.01	0.35	0.33
Length of eye	0.5	–	0.5	0.5
Ocellar tubercle width	0.9	–	0.9	0.9
Scape length	4.25	0.07	4.3	4.2
Pedicel length	1.35	0.07	1.4	1.3
Basiflagellomere length	5.25	0.07	5.3	5.2
Basiflagell. max. width	0.15	0.07	0.2	0.1
Distiflagellomere length (*n* = 1)^2^	–	–	1.5	1.5
Labial segment II length^1^	1.2	–	1.2	1.2
Labial segment III length^1^	0.9	–	0.9	0.9
Labial segment IV length^1^	0.25	0.07	0.3	0.2
Pronotum length (at midline)	2.65	0.07	2.7	2.6
Pronotum maximum width	2.9	–	2.9	2.9
Scutellum length	1.2	–	1.2	1.2
Fore femur length	3.65	0.07	3.7	3.6
Fore tibia length	4.1	0.14	4.2	4.0
Fore tarsus length	0.5	-	0.5	0.5
Middle femur length	3.1	0.14	3.2	3.0
Middle tibia length	3.85	0.07	3.9	3.8
Middle tarsus length (*n* = 1)^2^	–	–	0.8	0.8
Hind femur length	4.8	0.14	4.9	4.7
Hind tibia length	5.25	0.07	5.3	5.2
Hind tarsus length	0.65	0.07	0.7	0.6
Abdomen length^3^	7.1	0.14	7.2	7.0
Abdomen maximum width	1.85	0.07	1.9	1.8

^1^ Measured in lateral view; ^2^ Segments or portions broken or missing in some specimens; ^3^ Measured on ventral view, at midline, from anterior margin of sternite II to posterior border of genitalia.

##### Comments.

The genitalia of different males presenting a range of color variation (e.g. Figs 17–19) showed to have the same characteristics of structure (Figs 72–87). The females were slightly larger than the males (Tables 1, 2). The minimum body length in females (to tip of hemelytra/tip of abdomen: 15.1/12.0) is greater than the maximum body length in males (14.0/11.0). Many of the other measurements are proportionally greater in females, in accordance with their bigger size (Tables 1, 2), including the antennal scape and pedicel, slightly longer than in males. One apparent exception is the basiflagellomere, which was longer in most males (5.1 to 6.5 mm in length; *n* = 8) than in females (5.2 to 5.3 mm in length; *n* = 2) and showed to be generally thicker approximately in basal half in males (maximum width: 0.2–0.4 mm) (Figs 17–19, 38–40), but thinner in females (maximum width: 0.1–0.2 mm) (Figs 88, 94). This thickened region in males is completely covered by blackish, stiff, adpressed, and short setae (Figs 38–40), which are absent in females (Fig. 94). While the males presented a wide range of variation in coloration and markings, the two females examined presented different patterns of coloration, but similar to some of the males. Also, the patches of minute, short, adpressed, thin, whitish setae, covered with a variable amount of white wax, present on sternites IV–VII and even on the basal portion of the genitalia (Fig. 97), were absent in the males, with the exception of the record of a narrow stripe of whitish setae on distal half of sternite VII, extending to the basal portion of the exposed portion of pygophore in a single male (Fig. 69). However, because the relatively low number of specimens examined, especially of females (only two), it is not possible to be sure in what extent most of these differences are intraspecific or sexually dimorphic characteristics. Similarly, in relation to coloration, only the examination of more specimens of both sexes will allow ascertaining the range of variation and if there is any sexual dimorphism.

##### Distribution.

Brazil, in states of Minas Gerais and Paraná.

##### Etymology.

The new species is named in honor of Dr. Mara Cristina Pinto (Faculty of Pharmaceutical Sciences, UNESP, Araraquara, São Paulo, Brazil), a friend and eternal mentor of the second author (JO), as a tribute and recognition for her contributions to the studies of Medical Entomology, especially those on sandflies and also for all her meritorious performance as a teacher and knowledge as a transforming agent. The taxon’s homage is a way of rewarding all her remarkable contributions to Brazilian entomology which she has been studying for more than 33 years.

##### Comments.

The variation in color and number and extension of pale markings recorded among the specimens of *Q.maracristinae* sp. nov. studied here are considered as intra-specific variability. It is in accordance with the intraspecific variation in color, occasionally at extreme range, previously documented in many harpactorines (e.g., Stål 1872; Champion 1899; Gil-Santana 2008, 2022; Zhang et al. 2016), including in some wasp-mimicking Harpactorini (Champion 1899; Gil-Santana et al. 2013, 2017).

The wax-like substance was sometimes absent from portions where it was observed on other specimens. It may be lost during the manipulation of the individuals, which may also include loss of the thin fragile setae associated with it (HRG-S pers. obs.; Gil-Santana et al. 2017). Body parts covered with patches of setae with whitish wax-like material have been registered in some Harpactorini species, such as *Cosmoclopiuscuracavensis* Cobben & Wygodzinsky, 1975 (Cobben and Wygodzinsky 1975), *Harpactorangulosus* (Lepeletier & Serville, 1825) (Pikart et al. 2014), various species of *Heza* Amyot & Serville, 1843 (Maldonado 1976), *Sphedanolesteszhengi* Zhao, Ren, Wang & Cai, 2015 (Zhao et al. 2015), and *Parahiranetissalgadoi* (Gil-Santana et al. 2017). It is noteworthy that the wax-like substance may be absent when specimens are examined and described, and thus the extent of their existence may remain unknown (Gil-Santana et al. 2017). Similarly, records of the presence or absence of a wax-like substance may be an additional feature of systematic or taxonomic importance, in the same way as suggested for the “extensive sericeous areas on the abdominal sterna” of *Hezaventralis* Stål, 1872 (Maldonado 1976). Therefore, as stressed by Gil-Santana et al. (2017), future studies on Harpactorini should include careful handling of the specimens after collection, to avoid unintentional removal of these substances from their bodies. It is also recommended that this information should be included in the records and/or descriptions whenever present.

Differences in the structure and vestiture of the basiflagellomere were clear-cut enough to be considered sexually dimorphic in *Q.maracristinae* sp. nov. Despite the small number of females, adults can be sexed readily with the naked eye, by observing the basiflagellomeres of their antennae. The females examined were larger than males in many of the morphological characteristics measured, what can be confirmed by studying more specimens in the future. In any case, the two sexual differences pointed out in *Q.maracristinae* sp. nov. (i.e., females larger than males and the latter with basiflagellomere thickened) are concordant with several observations in the literature (Champion 1899; Martin-Park et al. 2012; Gil-Santana et al. 2013, 2017; Gil-Santana 2016). Additionally, the thickened portion of the basiflagellomere in males was completely covered by short, stiff, adpressed, blackish setae, which were absent in females. Although fewer females were examined, their coloration showed similar patterns of variation of some of the males, therefore only with the examination of more specimens will be possible to ascertain possible sexual variation in coloration patterns. Yet, in the females, patches of minute, short, adpressed, thin, whitish setae, covered with a variable amount of white wax, were present on sternites IV–VII and even on the basal portion of the genitalia, while in the males they were absent (with the exception of a single male in which only a narrow stripe of whitish setae was present on distal half of sternite VII, extending to the basal portion of the exposed portion of pygophore). In other wasp-mimicking harpactorines, such as *Parahiranetissalgadoi*, similar patches of setae covered with white wax on sternites were observed in both sexes (Gil-Santana et al. 2017). Therefore, it is necessary to examine more specimens in order to ascertain if the absence/presence of these patches on sternites in males and females of *Q.maracristinae* sp. nov., respectively, expresses another sexual dimorphism or if it is merely an inter-individual variation.

In the male genitalia, while the variation in color of the pygophore (Figs 67–72) is compatible with the general intra-specific variability in coloration, the uniformity of the other characteristics (Figs 72–87) is in accordance with the assumption that all specimens belong to the same species.

Yet, the male genitalia of *Q.maracristinae* sp. nov. showed similarities to those of *G.bicolor* (Gil-Santana et al. 2013), *H.atra* (Gil-Santana 2016), and *P.salgadoi* (Gil-Santana et al. 2017), such as: - parameres similar in shape and somewhat similar in vestiture; - pygophore with a somewhat large medial process that is medially rounded at the apex, but in *G.bicolor* and *H.atra* it is subtriangular in shape, while in *P.salgadoi* and *Q.maracristinae* sp. nov. it is somewhat arrow-shaped, with the lateral margins acutely pointed (Figs 72–74); - pedicel (pd) (= basal plate extension) short; - struts with subparallel median arms and curved basal lateral arms, although with different shapes in each species; - a pair of elongate, parallel, flat, weakly sclerotized endosomal processes, although with different locations and shapes in each of these species.

The presence of a somewhat laterally expanded basal portion with small acute spines on lateral margins of the dorsal phallothecal plate was recorded in *P.salgadoi* and *Q.maracristinae* sp. nov. U-shaped and median subspherical endosomal processes very similar to those of *Q.maracristinae* sp. nov. (Figs 80, 87) were recorded in *H.atra* and *P.salgadoi*. Yet, variable, different, or not well evident spiny lobes or portions of endosoma wall were recorded in each of these species, making their comparison difficult.

On the other hand, the general shape and peculiarities of the dorsal phallothecal plate were different in all species (Gil-Santana et al. 2013; Gil-Santana 2016; Gil-Santana et al. 2017; this study).

Thus, in agreement with previous studies (Elkins 1954a, b; Hart 1975, 1986, 1987; Forero et al. 2008; Zhang et al. 2016), the features of the male genitalia of *Q.maracristinae* sp. nov. that should especially be taken into consideration for comparative purposes are the shape of the medial process of the pygophore and the features of the dorsal phallothecal plate.

#### ﻿ Xystonyttus

Taxon classificationAnimaliaHemipteraReduviidae

Kirkaldy, 1909

2510A527-1820-5607-BE4E-487C5E2E1028

Cosmonyttus Stål, 1868: 103; Stål 1872: 83 (not Stål 1866: 295); Lethierry and Severin 1896: 178 [catalog; including erroneously Stål 1866: 295]. Type species: ZelusichneumoneusFabricius 1803: 286, by monotypy.
Xystonyttus Kirkaldy, 1909: 388 [as a new name for “Cosmonyttus, Stål, 1872 (not 1866)”]; Wygodzinsky 1949: 48 [catalog]; Putshkov and Putshkov 1985: 66 [catalog]; Maldonado 1990: 324 [catalog]; Maldonado and Lozada 1992: 165 [key]; Forero 2011: 16 [checklist]; Gil-Santana 2015: 37 [key], 2016: 92 [citations]; Gil-Santana et al. 2017: 41 [citation]. Type species: ZelusichneumoneusFabricius 1803: 286, by original designation.

##### Morphological remarks.

Head gibbous, large, as long as wide across eyes, densely covered with long setae on ventral and postocular portions; postantennal spines elongated, curved forward, apices acute. Legs: fore and hind femora curved at median portion; fore femora thickened, narrowing at apices; fore tibia curved at apical third; middle and hind legs elongated, slender. Hemelytra long, surpassing the abdomen by somewhat more than half of the length of the membrane.

#### ﻿ Xystonyttus ichneumoneus

Taxon classificationAnimaliaHemipteraReduviidae

(Fabricius, 1803)

9505484F-D094-5FD4-AA49-7777AA95F6B0

Figs 101–108

Zelus ichneumoneus Fabricius, 1803: 286 [description; “Habitat in America meridionali”].
Cosmonyttus ichneumoneus Stål, 1868: 103–104 [redescription; color varieties “a”, “b” and “c”; record from Suriname]; Stål 1872: 83 [catalog]; Lethierry and Severin 1896: 178 [catalog; cited as being from Guyana].
Myocoris ichneumoneus Walker, 1873b: 129 [catalog; record from Brazil].
Xystonyttus ichneumoneus Kirkaldy, 1909: 388 [type species to Xystonyttus, new name to Cosmonyttus Stål, 1872]; Haviland 1931: 150 [record from Guyana]; Wygodzinsky 1949: 48 [catalog; cited as being from [British] Guyana]; Maldonado 1990: 324 [catalog; cited as being originally described from [British] Guyana]; Gil-Santana et al. 2003: 12 [comment on its etymology].

##### Notes.

**Figures 101–108.**
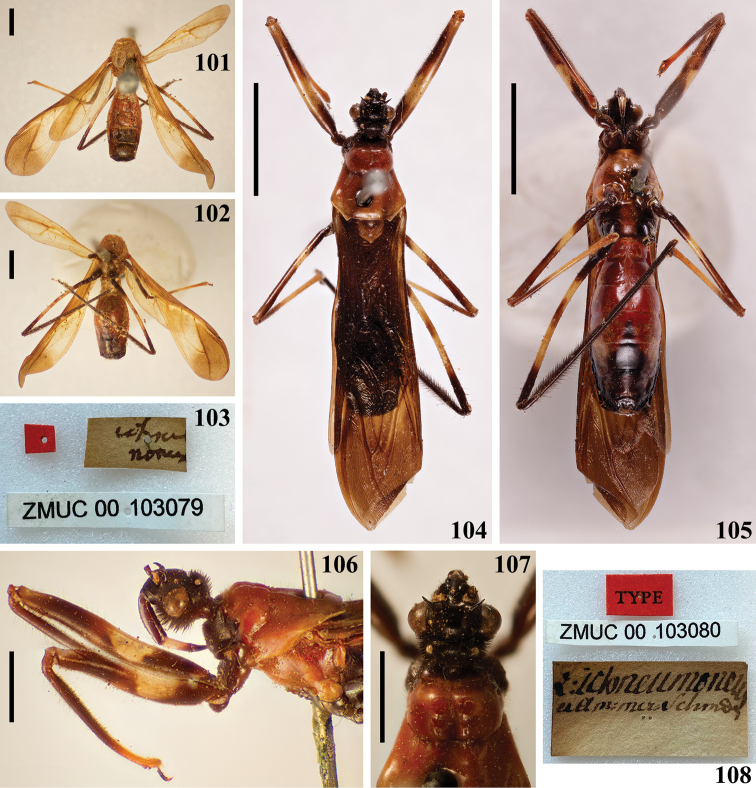
*Zelusichneumoneus* Fabricius, 1803, female syntypes deposited in ZMUC**101–103** syntype catalog number ZMUC 00 103079 **101** dorsal view **102** ventral view **103** labels **104–108** syntype catalog number ZMUC 00 103080 **104** dorsal view **105** ventral view **106** head, pronotum, fore legs, lateral view **107** head and most part of pronotum, dorsal view **108** labels. Scale bars: 5.0 mm (**104, 105**); 2.0 mm (**101, 102, 106, 107**).

The general similarity of *X.ichneumoneus* (Figs 101, 102, 104, 105) with ichneumonids certainly led Fabricius (1803) to name it as such (Gil-Santana et al. 2003). Fabricius (1803) recorded the species from South America (“America meridionali”) without specifying a country or location. The citations of Guyana (“British Guiana”) as the country from which the species was described, apparently were first stated by Lethierry and Severin (1896). Possibly, it was assumed because Fabricius (1803) cited “Dom. Smidt. Mus. Dom. de Sehestedt” when stating about the specimens of *Zelusichneumoneus* examined by him. In fact, “most of the numerous South American species that Fabricius described and for which a certain Smidt is cited as a collector are found only in the Copenhagen Museum, in Tönder Lund’s and Sehestedt’s famous collections (…). The only information (…) on Smidt is that he visited besides several West Indian islands, certain places on the South American mainland, such as Essequibo and Demerara in the present British Guiana; therefore all of the South American species cited as having been collected by Smidt can with certainty be considered as coming from the vicinity of the named localities, and this is just what one who is familiar with American Hemiptera and their distribution immediately perceives” (Stål 1868: 3, translation by G. C. Steyskal in Papavero 1971: 21). Stål (1868: 104), however, when recording the distribution of *X.ichneumoneus* maintained the original statement of Fabricius (1803) (“America meridionalis. Dom Smidt. (Mus. Sehestedt)”), adding: “Surinam. (Mus. Holm.)”. The latter refers to the specimen(s) examined and considered as belonging to *X.ichneumoneus* by himself in Stockholm Museum (currently, Swedish Museum of Natural History, Stockholm, Sweden), while Stål (1872) restricted the occurrence of the species to Suriname only. In any case, Haviland (1931) recorded *X.ichneumoneus* from Kartabo, Bartica District, Guyana, confirming the presence of the species in this country.

In the Natural History Museum of Denmark (ZMUC), Copenhagen, Denmark, there are two type specimens of *X.ichneumoneus*, both of them females, and considered here as syntypes, following Art. 73.2 of ICZN. In the syntype catalogued as ZMUC 00 103079, the head, fore legs, and a portion of the prothorax are missing (Figs 101, 102), while the other syntype, numbered as ZMUC 00 103080, is quite well preserved, with only the antennae and most tarsi missing (Figs 104–107). In the original label attached to the latter syntype (Fig. 108) the word “*ichneumoneus*” is clearly legible. Both specimens seem to belong to the same species judging by the remaining portions of the syntype ZMUC 00 103079 (Figs 101, 102), while the characteristics observed in the syntype ZMUC 00 103080 (Figs 104–107) agree very well with the original description (Fabricius 1803: 286). The photographs presented here are helpful to ascertain not only the characteristics of the species but also those regarded as establishing limits between the recognized genera of Neotropical wasp-mimicking Harpactorini.

The concise description of the coloration of the specimens of *X.ichneumoneus*, as recorded by Haviland (1931), without mention of any variation, was quite similar not only with the original description of the species (Fabricius 1803) but also with that observed in the syntypes (Figs 101, 102, 104–107). The color variations attributed to *X.ichneumoneus* by Stål (1866), however, are in need to be reviewed by examining series of specimens with more comprehensive approaches, since they may be variations of this single species or may represent two or more different species.

## ﻿Discussion

Among the Neotropical Harpactorini, *Quasigraptocleptes* gen. nov. seems closer to *Graptocleptes* and *Hiranetis*, while the latter have been considered allied genera (Stål 1872; Champion 1899) and also close to *Parahiranetis* (Gil-Santana 2015; Gil-Santana et al. 2017).

The main diagnostic characteristics that separate *Hiranetis*, *Graptocleptes*, *Parahiranetis* and *Quasigraptocleptes* gen. nov. according to Spinola (1840a, b), Stål (1866, 1872), Maldonado and Lozada (1992), Gil-Santana et al. (2013, 2017), Gil-Santana (2015, 2016) and this work, are the following:

1. *Graptocleptes*: head elongate, approximately 1.1 to 1.3 times as long as width across eyes; legs thicker; fore femur shorter than head and pronotum together, somewhat thicker basally or with uniform thickness.
2. *Hiranetis*: head gibbous, swollen ventrally, approximately as long as width across eyes; legs elongated, slender; fore femur subequally longer than head and pronotum together, thicker basally.
3. *Parahiranetis*: head elongate, approximately 1.3–1.7 times as long as width across eyes; legs elongated, slender; fore femur subequally longer than head and pronotum together, thicker basally.
4. *Quasigraptocleptes* gen. nov.: head gibbous, swollen ventrally, approximately as long as width across eyes; legs thicker; fore femur shorter than head and pronotum together, somewhat thicker basally.

Additionally, while *Quasigraptocleptes* gen. nov. has conspicuous postantennal spines strongly curved backwards and completely or partially directed medially (Figs 20–33), in most species of the other three genera mentioned above, these spines are absent or present as small postantennal tubercles or in some species of *Graptocleptes* as straight vertical or semivertical spines (Stål 1872; Maldonado and Lozada 1992; Gil-Santana et al. 2013, 2017; Gil-Santana 2015, 2016, 2022).

Historically, only the pattern of yellowish or straw-colored hemelytra with a median transverse black band has received attention in regard to the supposed mimicry between Harpactorini and species of hymenopteran Ichneumonidae and Braconidae (Champion 1899; Haviland 1931; Maldonado and Lozada 1992; Hogue 1993; Leathers and Sharkey 2003; Hespenheide 2010). However, some species that have similar patterns of blackish wings with yellowish ‘pterostigmata’ and pale bands on the middle and hind femora arise as possible candidates for mimetic complexes, including *Q.maracristinae* sp. nov. As suggested by Gil-Santana et al. (2017), these would be the wasp-mimicking harpactorines *Graptocleptesbicolor*, *G.haematogaster* (Stål, 1860), an undescribed species of *Hiranetis* and *Parahiranetissalgadoi* as well as ichneumonoid wasps and a species of the cerambycid beetle, all of them recorded from southeastern Brazil.

Although there are records of color variation in some wasp-mimicking Harpactorini, at least in the species with the pattern of darkened or blackish hemelytra with yellowish pterostigmata, there is no variation in this pattern. The yellowish pterostigmata are always present (e.g., Gil-Santana et al. 2013, 2017). The specimens of *Q.maracristinae* sp. nov. studied here were concordant with this assumption, taking into account that, in spite of a considerable range of variation in color, the mentioned pattern of the hemelytra was present in all of them. Additionally, as emphasized by Gil-Santana (2015) and Gil-Santana et al. (2017), it is necessary to elucidate which species or groups of insects share the same color pattern as in *Q.maracristinae* sp. nov. (i.e., blackish to reddish coloration with yellowish ‘pterostigmata’ on wings and/or yellowish markings on legs) are involved in possible mimicry complexes.

### ﻿Key to the Neotropical genera of wasp-mimicking Harpactorini

Based on Stål 1866, 1872; Elkins 1969; Maldonado and Lozada 1992; Gil-Santana 2015; Castro-Huertas and Forero 2021.

**Table:** 

1	Pronotum greatly inflated and covering scutellum posteriorly	***Coilopus* Elkins, 1969**
–	Pronotum not inflated; scutellum not covered by the posterior portion of pronotum and visible from above	**2**
2	Hind lobe of pronotum with a pair of elevated submedial longitudinal carinae and spines on its posterior margin; fore trochanter with a ventral spine in most species	***Acanthischium* Amyot & Serville, 1843**
–	Hind lobe of pronotum different; fore trochanter without spines	**3**
3	Postantennal spines curved and directed forward	**4**
–	Postantennal spines absent, as tubercles, straight directed vertically, semivertically or strongly curved backwards	**6**
4	Head generally sparsely setose (Figs 3, 11, 14)	***Myocoris* Burmeister, 1835**
–	Head quite setose to very densely setose, especially on ventral and post-ocular portions	**5**
5	Fore femora almost entirely thickened, somewhat narrowing at apex only; fore tibiae curved at apical third (Fig. 106)	***Xystonyttus* Kirkaldy, 1909**
–	Fore femora thicker only basally; fore tibiae straight	***Neotropiconyttus* Kirkaldy, 1909**
6	Postantennal spines strongly curved backwards and completely or partially directed medially (Figs 20–33)	***Quasigraptocleptes* gen. nov.**
–	Postantennal spines absent, or straight directed vertically, semivertically, or as tubercles	**7**
7	Fore femora slender, elongated; clearly thicker basally	**8**
–	Fore femora stouter; not or only slightly thicker basally	***Graptocleptes* Stål, 1866**
8	Head gibbous, swollen ventrally, subequally as long as wide across eyes; in dorsal view, postocular portion clearly separated from a distinct neck	***Hiranetis* Spinola, 1837**
–	Head elongate, not swollen ventrally, approximately 1.3 to 1.7 times as long as wide across eyes; in dorsal view, postocular portion narrowing gradually to form neck	***Parahiranetis* Gil-Santana, 2015**

## Supplementary Material

XML Treatment for Myocoris

XML Treatment for Myocoris nigriceps

XML Treatment for Myocoris nugax

XML Treatment for Myocoris tipuliformis

XML Treatment for Quasigraptocleptes

XML Treatment for Quasigraptocleptes maracristinae

XML Treatment for Xystonyttus

XML Treatment for Xystonyttus ichneumoneus
